# A Global View of the Relationships between the Main Behavioural and Clinical Cardiovascular Risk Factors in the GAZEL Prospective Cohort

**DOI:** 10.1371/journal.pone.0162386

**Published:** 2016-09-06

**Authors:** Pierre Meneton, Cédric Lemogne, Eléonore Herquelot, Sébastien Bonenfant, Martin G. Larson, Ramachandran S. Vasan, Joël Ménard, Marcel Goldberg, Marie Zins

**Affiliations:** 1 INSERM U1142 LIMICS, UMR_S 1142 Sorbonne Université, UPMC Université Paris 06, Université Paris 13, Paris, France; 2 Centre Psychiatrie et Neurosciences, INSERM U894, Université Paris Descartes, AP-HP Hôpitaux Universitaires Paris Ouest, Paris, France; 3 INSERM UVSQ UMS 011 and UMR-S 1168 VIMA, Villejuif, France; 4 Department of Biostatistics, Department of Mathematics and Statistics, Boston University, Boston, MA, United States of America; 5 Framingham Heart Study, Department of Medicine, Boston University, Boston, MA, United States of America; 6 INSERM/AP-HP CIC1418, Université Paris Descartes, AP-HP Hôpitaux Universitaires Paris Ouest, Paris, France; Heinrich-Heine-Universitat Dusseldorf Medizinische Fakultat, GERMANY

## Abstract

Although it has been recognized for a long time that the predisposition to cardiovascular diseases (CVD) is determined by many risk factors and despite the common use of algorithms incorporating several of these factors to predict the overall risk, there has yet been no global description of the complex way in which CVD risk factors interact with each other. This is the aim of the present study which investigated all existing relationships between the main CVD risk factors in a well-characterized occupational cohort. Prospective associations between 12 behavioural and clinical risk factors (gender, age, parental history of CVD, non-moderate alcohol consumption, smoking, physical inactivity, obesity, hypertension, dyslipidemia, diabetes, sleep disorder, depression) were systematically tested using Cox regression in 10,736 middle-aged individuals free of CVD at baseline and followed over 20 years. In addition to independently predicting CVD risk (HRs from 1.18 to 1.97 in multivariable models), these factors form a vast network of associations where each factor predicts, and/or is predicted by, several other factors (n = 47 with p<0.05, n = 37 with p<0.01, n = 28 with p<0.001, n = 22 with p<0.0001). Both the number of factors associated with a given factor (1 to 9) and the strength of the associations (HRs from 1.10 to 6.12 in multivariable models) are very variable, suggesting that all the factors do not have the same influence within this network. These results show that there is a remarkably extensive network of relationships between the main CVD risk factors which may have not been sufficiently taken into account, notably in preventive strategies aiming to lower CVD risk.

## Introduction

Over the last decades, many risk factors have been independently associated with cardiovascular diseases (CVD) through prospective observational studies [1, 2]. This includes behavioural risk factors (smoking [3], alcohol consumption [4] or physical inactivity [5]), biological characteristics (gender [6], age [7] or familial history of CVD [8]), clinical traits reflecting the progression of CVD (hypertension [9], dyslipidemia [10], obesity [11] or diabetes [12]), psychological factors (job strain [13], sleep disorders [14, 15] or depression [16]), and more recently biochemical markers [17] and genetic polymorphisms [18].

Despite the recognition that these factors do not act separately but jointly to determine CVD risk [19] and the fact that several of them have been combined in various ways to calculate scores aimed to predict the overall risk [20], most often they have been studied individually at different times or in different cohorts, the other factors being used mainly for statistical adjustment purposes. This makes difficult to compare the relative effect of each factor and it does not facilitate the appraisal of the network of interactions between CVD risk factors [21]. Two notable exceptions are the INTERHEART [22] and INTERSTROKE [23] studies which have simultaneously assessed a significant number of CVD risk factors with a transversal case-control approach. These studies have provided insights about nine or ten factors independently associated with the risk of coronary heart disease or stroke and accounting altogether for the quasi-totality of the overall risk. However, even in these two studies, the relationships between the risk factors themselves were not specifically assessed. This is also the case of a recently published study which has performed a systematic comparison of the predictors of CVD mortality in the UK Biobank population during a 5-year period [24].

The interest of taking into account the relationships between the risk factors is potentially high, not so much for the identification of individuals with an increased CVD risk that is usually quite good [25] but for improving the efficiency of preventive strategies aiming to lower this risk which are notoriously ineffective [26, 27]. Among the multiple reasons for this ineffectiveness, the failure of sufficiently considering the relationships between the risk factors may be an important one. Indeed, the simultaneous presence of several factors can lead to partial or contradictory interventions if these factors reinforce themselves by interacting with each other.

As a first step to take into account this interaction network, the present study aims to provide a global view of all the prospective relationships between the main behavioural and clinical CVD risk factors in a single cohort followed over a 20-year period.

## Methods

### Study population

The analyses were performed in a cohort of middle-aged volunteers working at the French National Gas and Electricity Company, who were all recruited in 1989 and followed since then (GAZEL cohort) [28]. The recruitment took place after an information campaign inviting all men and women aged 35–50 years to participate in the cohort on a voluntary basis. The workers who volunteered (n = 20,625) were mainly white individuals of European ancestry and lived throughout the French metropolitan territory in various settings ranging from rural areas to urban centres, they have been shown to be very diverse in terms of their social, economic and occupational status, health and health-related behaviours [29]. They were very motivated to participate in the cohort study as indicated by the high acceptance rate at the time of the recruitment (45%) and the very low attrition rate during follow-up (~ 3%). The response rate to a self-administered annual questionnaire also remained high during the entire follow-up (around 75%) with only less than 5% of the initial cohort who never sent back any questionnaire. All the volunteers sent written informed consent to participate to the study which has received approval from the Ethics Evaluation Committee of the Institut National de la Santé et de la Recherche Médicale and the National Committee for the Protection of Privacy and Civil Liberties (IRB0000388, FWA00005831).

### Assessment of risk factors and CVD events

All the data were obtained from self-administered questionnaires, completed annually by volunteers during follow-up, which contained a variety of inquiries into their lifestyle and the occurrence of health events. Among the risk factors that were considered in the analyses, gender and age were reported as such. Parental history of CVD referred to the occurrence of coronary heart disease before the age of 60 in the mother or the father. The inquiry into the occurrence of hypertension, dyslipidemia, diabetes, sleep disorder and nonfatal CVD events (which included angina pectoris, myocardial infarction, stroke and arteritis of lower limbs) was composed of two consecutive questions: first, “in the list below, indicate the diseases you are suffering or have suffered from during the past twelve months” and secondly, “among the diseases you have cited, what are the new ones, i.e., those you were not suffering from over a year ago”? Body mass index was calculated from weight and height values drawn from the questionnaires. Depressive status was assessed with the Centre of Epidemiologic Studies Depression scale and defined as scores ≥ 17 in men and ≥ 23 in women, which includes both moderate and severe depression [30]. The inquiry into alcohol consumption and smoking referred to the habits during the week before completing the questionnaires and was designed to take into account the culture of French people who consume alcohol on a regular basis [31]. Physical activity was defined by the practice of a sport whatever its frequency (occasionally, regularly or competition) as opposed to physical inactivity (no sport practice).

As the validity of self-reporting for assessing the incidence of risk factors and CVD events is always questionable, a quality control study has been performed in the GAZEL cohort by comparing CVD disorders that were reported in the questionnaire in 1992 with physician’s diagnoses obtained from the sick-leave database of the company [32]. It shows that the issue is not over-reporting but under-reporting with a rate depending on the disorder, the characteristics of the individuals and the methodology used. Thus, under-reporting rate varies substantially from one CVD disorder to another (95.2% for diabetes, 80.4% for hypertension, 77.8% for arteritis of lower limbs, 72.4% for myocardial infarction, 71.4% for angina pectoris, 54.5% for stroke).

### Statistical analyses

Twelve clinical or behavioural factors were used to predict the risk of CVD events during follow-up. The rationale behind this choice was that these traditional risk factors are commonly used by physicians in their daily practice where the prevention may take place and they are likely to be available in cohorts in which the present analyses could be reproduced. Gender, parental cardiovascular disease, physical activity, hypertension, dyslipidemia, diabetes, sleep disorder and depression were coded as binary variables (women/men for gender, no/yes for the others). Age (range 39–54 years) was divided into tertiles and body mass index (BMI) into the usual 3 categories: optimal (BMI < 25 kg/m^2^), overweight (25 kg/m^2^ ≤ BMI < 30 kg/m^2^) or obese (BMI ≥ 30 kg/m^2^). Smoking was classified into 3 groups (non-smoker, ex-smoker, smoker) and alcohol consumption into 4 patterns using a slightly modified version of the World Health Organisation classification to take into account the distribution of the consumption in the French population: non-drinker, light drinker (1–13 drinks/week in men, 1–6 drinks/week in women), moderate drinker (14–27 drinks/week in men, 7–20 drinks/week in women) or heavy drinker (≥ 28 drinks/week in men, ≥ 21 drinks/week in women). Except for gender, age and parental CVD, the incidence of each factor during follow-up was also considered as an outcome that can be predicted by the other factors at baseline. In this case, physical inactivity, hypertension, dyslipidemia, diabetes, sleep disorder and depression were used as such. Body mass index, smoking and alcohol consumption were transformed into binary variables as follows: obese versus optimal/overweight, smoker versus non-smoker/ex-smoker and non-moderate drinker (non-drinker/light drinker/heavy drinker) versus moderate drinker.

Cox proportional hazard regression models (unadjusted, adjusted for gender and age, and multi-adjusted for the 12 risk factors (except the one being tested)) were used to compute hazard ratios and 95% confidence intervals for incident CVD events and for the incidence of each risk factor during a 20-year follow-up running from 1993 to 2013. The baseline year was 1993 because it was the first year in which all the risk factors were simultaneously assessed. Time to event was measured from baseline to the first occurrence of CVD events or the first detection of the risk factors as provided by the annual questionnaires. For each factor, the time-dependent number of volunteers at risk analysed in the corresponding Cox regression is shown in **S1 Fig.** The proportionality of hazards was verified by using stratified Kaplan-Meier curves which show graphically (curves roughly parallel after log(-log(survival)) versus log(time) transformation) that the assumption holds for the 47 significant associations reported in the results (**S2 Fig**). Note that the assumption was not verified for the 52 non-significant associations as the identification of those violating the assumption would not allow to include them in the network of significant associations but only to suspect them to be false negatives. Despite the fact that annual questionnaires produced interval censored data in the unit of one year, standard Cox regressions were used in place of proportional hazard regressions for interval censored data because it was assumed that the failure to report incident CVD events or risk factors or the delay with which they are reported in the following questionnaires do not greatly modify the estimation of hazard ratios as there is no evidence in the GAZEL cohort that the failure frequency or delay length vary to a large extent between the volunteers exposed and those not exposed to the risk factors.

Censored events include all deaths (n = 707) among which some were of cardiovascular origin (n = 112). This certainly leads to an underestimation of the magnitude of the associations with nonfatal CVD events as fatal and nonfatal events are supposed to have the same risk factors. However, this underestimation remains minimal given that the proportion of fatal events in comparison to non-fatal events is very low (7.8%). The reason of this choice is that the cause of death was not sufficiently documented to allow an accurate assessment of fatal events. Note that when analysing the incidence of risk factors, volunteers who have had a nonfatal CVD event before the first detection of the factors were also censored. Although the mortality rate is relatively low (6.6% over follow-up, see **S3 Fig**), it is not negligible when compared to the incident rates of CVD events or of the risk factors, especially towards the end of the follow-up. Censoring deaths as the events of interest might therefore bias the observed associations to some extent. Nevertheless, Cox regressions were used in place of competing risk models for two reasons. The first one is that the main purpose of the study is to explore the presence or absence of relationships between the risk factors rather than to determine accurately their incidence. It has been shown that censoring competing events is acceptable in this case as the HRs are relatively similar in standard Cox regressions and competing risk methods [33]. The second reason is that, in the present study, all the risk factors that predict the appearance of CVD events or of the other risk factors also predict death. Thus, a potential bias would likely be an under-estimation and not an over-estimation of the HRs, thereby reducing the risk of creating false positives.

The risk of CVD events was assessed in 10,736 of the 20,625 volunteers originally included in the cohort after exclusion of those who have had a nonfatal CVD event before the beginning of the follow-up period (n = 247) or those who had missing data in one or more of the risk factors included in the regression models (n = 9778). Additionally, when the outcome was the incidence of CVD risk factors during follow-up, volunteers who were exposed to the risk factors before the follow-up period or at baseline were also excluded before performing the analyses (n = 163 to 8282 depending on the factor). Note that as volunteers lost to follow-up were among those who were excluded due to missing values, all the volunteers included in the analyses were followed over the whole period of 20 years except when they died during this time period. These analyses can therefore be seen as a retrospective study conditional on the follow-up period or death within this period. Although the number of volunteers lost to follow-up is very low (n = 619), a sensitivity analysis was made to test whether the exclusion of these volunteers may bias the results of the analyses. For this purpose, multi-adjusted Cox regressions including volunteers lost to follow-up were performed with a follow-up running up to the date of the last available questionnaire (**S1 Table**). The comparison with multi-adjusted Cox regressions excluding these volunteers with the 20-year follow-up indicates that the bias is very small as all the associations with incident CVD events and the vast majority of those with incident risk factors are conserved (one new association and none lost out of 47 at p<0.05, one new association and 3 lost out of 22 at p<0.0001).

The choice of using only volunteers with a complete set of data was made by considering that a multiple imputation approach in a cohort where the distribution of missing values is not monotonous (see the overall pattern of missing data for the 12 risk factors included in the analyses in **S4 Fig**) would require to use multivariate imputation by chained equations which is time-consuming and not always devoid of biases [34, 35]. Of course, the exclusion of a large number of volunteers with missing data leads to the selection of a significantly healthier population. As shown in **S2 Table**, the rates of hypertension, dyslipidemia, diabetes and depression, as well as those of smoking and physical inactivity, are significantly lower in the selected volunteers. To test to which extent this selection may affect the results presented in this study, two types of sensitivity analyses were performed. The first one was to run Cox regressions weighted by the inverse probability of being selected; this probability was calculated by using a multivariate logistic regression model including five risk factors, averaged over the 4-year period (1989–1992) preceding the baseline year in order to minimize the number of missing values, which were significantly associated with the selection (OR (95%CI): gender W/M 0.78 (0.71–0.87), hypertension Y/N 0.84 (0.75–0.94), dyslipidemia Y/N 0.81 (0.68–0.98), depression Y/N 0.71 (0.64–0.78) and smoking Y/N 0.77 (0.71–0.84). The comparison of the outputs of weighted (**S3 Table**) and unweighted multi-adjusted Cox regressions shows that all the associations with incident CVD events and most of those with incident risk factors are conserved (9 new associations and 3 lost out of 47 at p<0.05, 3 new associations and 2 lost out of 22 at p<0.0001). The second sensitivity analysis consisted of comparing the outputs of gender-age adjusted Cox regressions in volunteers with complete data either for all the risk factors (i.e., the population selected for the study) or only for the factor analysed in each model (given that gender and age were available for all volunteers); the number of volunteers excluded is large in the first case while it is much smaller and varies from one model to another depending on the risk factor in the second case. **S4 Table** shows that all the associations with incident CVD events and most of those with incident risk factors are conserved (12 new associations and none lost out of 61 at p<0.05, 10 new associations and 2 lost out of 36 at p<0.0001); note that the number of gender-age adjusted associations in volunteers with complete data in all the risk factors is greater than the number reported in the Results where only the associations observed in multi-adjusted models are shown. In both sensitivity analyses, it appears that the variations in the HRs (95% CI) explaining the shift of some associations above or below statistical thresholds are small in the vast majority of the cases.

**Fig 1:**
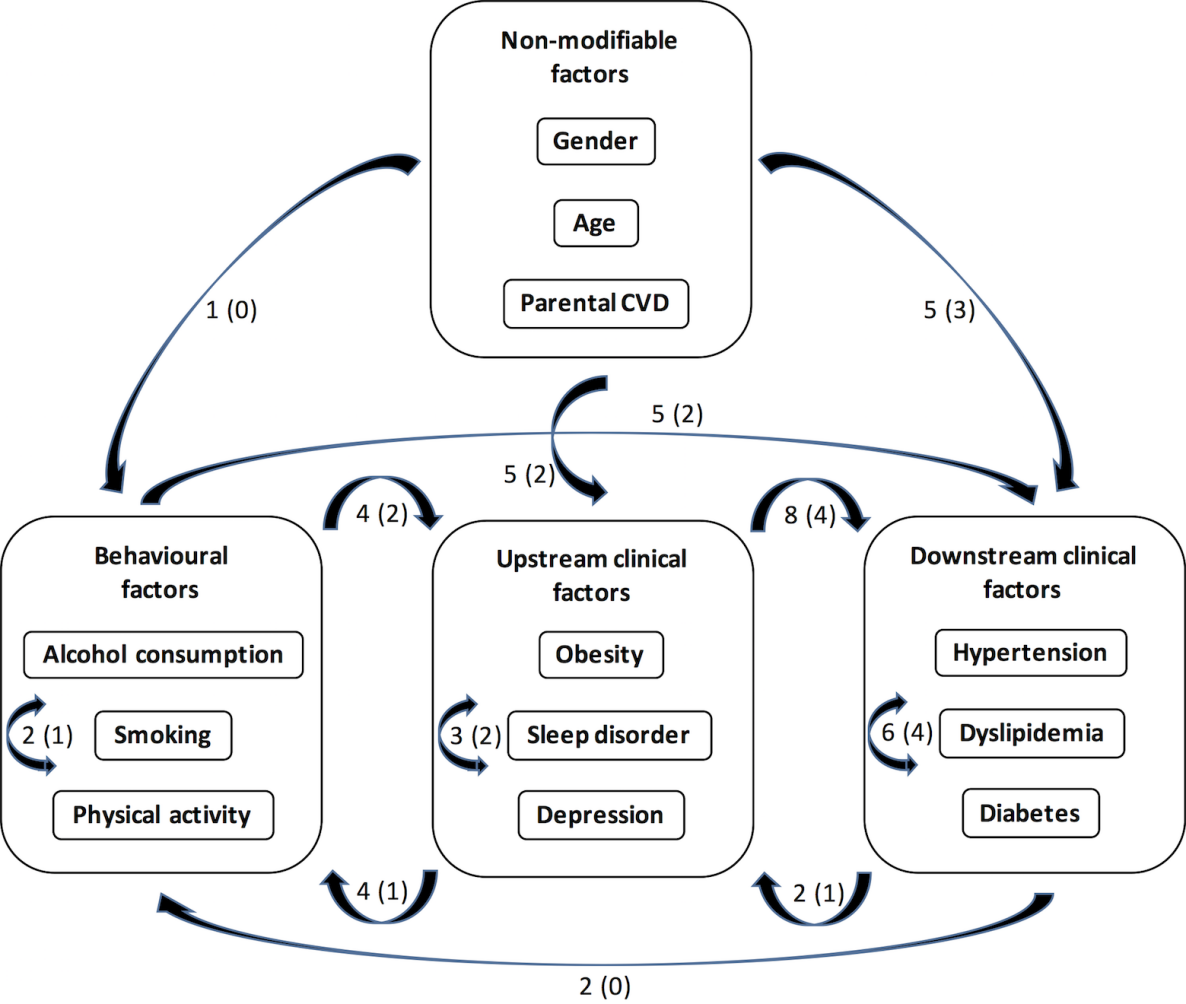
Conceptual framework of the relationships between CVD risk factors. The factors are grouped into 4 types based on the number of other factors predicting each of them. The numbers next to the arrows represent the number of prospective associations between or within the 4 types of factors at p<0.05 or p<0.0001 (in parenthesis).

Several risk factors such as hypertension, dyslipidemia or diabetes have a tendency to be irreversible once declared but others such as behavioural factors can potentially vary significantly throughout the 20-year follow-up. Multi-adjusted Cox regressions with time-dependent predictive risk factors (tested as binary covariates) were therefore performed to estimate the bias that may be introduced by considering these factors as time-independent. As shown in **S5 Table**, although the magnitude of some HRs can vary substantially (in the case of the associations with depression for example), all but one (physical inactivity) of the associations with incident CVD events and most of those with incident risk factors are conserved (3 new associations and 3 lost out of 47 at p<0.05, one new association and 4 lost out of 22 at p<0.0001). Most importantly, this does not modify the conceptual framework summarizing the main findings of the study (Fig 1) whether it is based on the associations at p<0.05 or on those at p<0.0001. Consequently, predictive risk factors have been considered as time-independent in the regression models presented in the Results for the sake of simplicity.

An issue related to multiple testing is the risk of having false positives which increases with the number of tests performed (n = 17 × 9 × 3 in the present case). To take this problem into account, a Bonferroni correction could be applied in order to keep only the tests with p<0.0001 (0.05/(17 × 9 × 3)). However, this correction comes at the cost of increasing the probability of having false negatives, which would be clearly the case in this study where several well-known dose-dependent associations are observed at 0.0001<p<0.05. Thus, rather than choosing between keeping potential false positives or excluding potential false negatives, we decided to show all the associations with p<0.05 leaving open the possibility of narrowing the significance threshold to 0.01, 0.001 or 0.0001, depending on the priority of the reader. In fact, this issue is not critical as the main conclusions that can be drawn from the analyses, i.e., the existence of an extensive network of relationships between the main CVD risk factors, its conceptual framework and its potential interest in prevention, are fundamentally the same whatever the significance threshold which is selected.

All analyses were performed with the statistical discovery software JMP Pro 12 (SAS, Cary NC).

## Results

### Associations of the risk factors at baseline with the incidence of CVD events

Among the 10,736 volunteers retained for the analyses, whose baseline characteristics are shown in **Table 1**, a total of 1694 nonfatal CVD events occurred during follow-up (mean/median 11.4/12.0 years for volunteers with event, 19.7/20.0 years for volunteers without event) corresponding to 7.9 events per 1000 person-years. The distribution of these events according to the risk factors at baseline is shown in **Table 2**with the hazard ratios. All the factors independently predict CVD risk with multi-adjusted HRs varying from 1.18 to 1.97.

**Table 1:** Baseline distribution of clinical or behavioural predictive factors among the 10736 volunteers retained to assess CVD risk.

		N (%)
**Gender**	Women	2723 (25.4)
	Men	8013 (74.6)
**Age (y)**	39–45	3542 (33.0)
	46–49	3547 (33.0)
	50–54	3647 (34.0)
**Parental CVD**	No	9437 (87.9)
	Yes	1299 (12.1)
**Alcohol consumption**	Non-drinker	1277 (11.9)
	Light drinker	5659 (52.7)
	Moderate drinker	2454 (22.9)
	Heavy drinker	1346 (12.5)
**Smoking**	Non-smoker	4694 (43.7)
	Ex-smoker	4035 (37.6)
	Smoker	2007 (18.7)
**Physical activity**	No	3365 (31.3)
	Yes	7371 (68.7)
**Body mass index**	Optimal	5762 (53.7)
	Overweight	4328 (40.3)
	Obesity	646 (6.0)
**Hypertension**	No	9717 (90.5)
	Yes	1019 (9.5)
**Dyslipidemia**	No	9030 (84.1)
	Yes	1706 (15.9)
**Diabetes**	No	10573 (98.5)
	Yes	163 (1.5)
**Sleep disorder**	No	7937 (73.9)
	Yes	2799 (26.1)
**Depression**	No	8314 (77.4)
	Yes	2422 (22.6)

**Table 2:** Risk of CVD events according to predictive factors at baseline.

		No. of cases	Unadjusted		Gender & age-adjusted		Multi-adjusted	
			HR (95% CI)	p	HR (95% CI)	p	HR (95% CI)	p
**Diabetes**	No	1638	1.00		1.00		1.00	
	Yes	56	2.65 (2.01–3.43)	<0.0001	2.36 (1.79–3.05)	<0.0001	1.97 (1.49–2.56)	<0.0001
**Gender**	Women	236	1.00		1.00		1.00	
	Men	1458	2.21 (1.93–2.54)	<0.0001	1.98 (1.72–2.28)	<0.0001	1.87 (1.61–2.18)	<0.0001
**Smoking**	Non-smoker	553	1.00		1.00		1.00	
	Ex-smoker	710	1.56 (1.39–1.74)	<0.0001	1.34 (1.20–1.50)	<0.0001	1.28 (1.14–1.44)	<0.0001
	Smoker	431	2.02 (1.78–2.29)	<0.0001	1.84 (1.62–2.09)	<0.0001	1.81 (1.59–2.06)	<0.0001
**Age (y)**	39–45	559	1.00		1.00		1.00	
	46–49	559	1.51 (1.33–1.72)	<0.0001	1.30 (1.14–1.48)	<0.0001	1.26 (1.10–1.44)	0.0006
	50–54	576	1.93 (1.71–2.19)	<0.0001	1.67 (1.47–1.89)	<0.0001	1.58 (1.40–1.80)	<0.0001
**Hypertension**	No	1437	1.00		1.00		1.00	
	Yes	257	1.87 (1.63–2.13)	<0.0001	1.77 (1.55–2.02)	<0.0001	1.52 (1.32–1.74)	<0.0001
**Body mass index**	Optimal	748	1.00		1.00		1.00	
	Overweight	779	1.43 (1.29–1.58)	<0.0001	1.20 (1.08–1.33)	0.0007	1.09 (0.98–1.21)	0.10
	Obesity	167	2.18 (1.83–2.57)	<0.0001	1.90 (1.60–2.25)	<0.0001	1.47 (1.23–1.75)	<0.0001
**Parental CVD**	No	1417	1.00		1.00		1.00	
	Yes	277	1.47 (1.29–1.67)	<0.0001	1.52 (1.34–1.73)	<0.0001	1.45 (1.28–1.65)	<0.0001
**Alcohol consumption**	Non-drinker	197	1.00		1.00		1.00	
	Light drinker	879	0.98 (0.85–1.15)	0.84	0.83 (0.71–0.97)	0.02	0.86 (0.74–1.01)	0.06
	Moderate drinker	358	0.93 (0.78–1.10)	0.39	0.75 (0.63–0.90)	0.002	0.75 (0.63–0.90)	0.002
	Heavy drinker	260	1.30 (1.08–1.56)	0.006	0.97 (0.80–1.17)	0.73	0.85 (0.70–1.03)	0.10
**Sleep disorder**	No	1189	1.00		1.00		1.00	
	Yes	505	1.23 (1.11–1.36)	0.0001	1.34 (1.21–1.49)	<0.0001	1.26 (1.13–1.40)	<0.0001
**Dyslipidemia**	No	1320	1.00		1.00		1.00	
	Yes	374	1.60 (1.43–1.79)	<0.0001	1.42 (1.26–1.59)	<0.0001	1.24 (1.10–1.39)	0.0004
**Physical activity**	No	623	1.00		1.00		1.00	
	Yes	1071	0.75 (0.68–0.83)	<0.0001	0.72 (0.66–0.80)	<0.0001	0.81 (0.73–0.90)	<0.0001
**Depression**	No	1237	1.00		1.00		1.00	
	Yes	457	1.32 (1.18–1.47)	<0.0001	1.34 (1.20–1.49)	<0.0001	1.18 (1.06–1.32)	0.004

### Associations between the risk factors at baseline and their incidence during follow-up

Only one risk factor independently predicts the incidence of non-moderate alcohol consumption (2477 cases among 2691 volunteers during follow-up (mean/median 2.9/1.0 years for volunteers with incident cases, 18.2/20.0 years for volunteers without incident cases) or 46.0 cases per 1000 person-years) with multi-adjusted HRs ranging from 1.13 to 1.14 (**Table 3)**.

**Table 3:** Risk of non-moderate alcohol consumption according to predictive factors at baseline.

		No. of cases	Unadjusted		Gender & age-adjusted		Multi-adjusted	
			HR (95% CI)	p	HR (95% CI)	p	HR (95% CI)	p
**Smoking**	Non-smoker	933	1.00		1.00		1.00	
	Ex-smoker	991	1.16 (1.06–1.26)	0.001	1.12 (1.02–1.23)	0.01	1.13 (1.03–1.24)	0.01
	Smoker	553	1.17 (1.05–1.30)	0.004	1.14 (1.02–1.27)	0.02	1.14 (1.02–1.27)	0.02

Only predictive factors significantly associated with incident non-moderate alcohol consumption in the multi-adjusted model are shown in the table.

The incidence of smoking (1041 cases among 8092 volunteers during follow-up (mean/median 8.3/7.0 years for volunteers with incident cases, 19.9/20.0 years for volunteers without incident cases) or 6.4 cases per 1000 person-years) is independently predicted by two factors with multi-adjusted HRs varying from 1.18 to 1.29 (**Table 4**).

**Table 4:** Risk of smoking according to predictive factors at baseline.

		No. of cases	Unadjusted		Gender & age-adjusted		Multi-adjusted	
			HR (95% CI)	p	HR (95% CI)	p	HR (95% CI)	p
**Body mass index**	Optimal	508	1.00		1.00		1.00	
	Overweight	466	1.29 (1.13–1.46)	<0.0001	1.29 (1.13–1.48)	0.0001	1.29 (1.12–1.47)	0.0002
	Obesity	67	1.25 (0.96–1.60)	0.09	1.26 (0.97–1.62)	0.08	1.27 (0.97–1.64)	0.08
**Depression**	No	786	1.00		1.00		1.00	
	Yes	255	1.19 (1.03–1.37)	0.02	1.19 (1.03–1.37)	0.02	1.18 (1.02–1.37)	0.02

Only predictive factors significantly associated with incident smoking in the multi-adjusted model are shown in the table.

Six factors independently predict the incidence of physical inactivity (3325 cases among 7371 volunteers during follow-up (mean/median 8.7/9.0 years for volunteers with incident cases, 19.2/20.0 years for volunteers without incident cases) or 22.6 cases per 1000 person-years) with multi-adjusted HRs ranging from 1.14 to 1.73 (**Table 5**).

**Table 5:** Risk of physical inactivity according to predictive factors at baseline.

		No. of cases	Unadjusted		Gender & age-adjusted		Multi-adjusted	
			HR (95% CI)	p	HR (95% CI)	p	HR (95% CI)	p
**Body mass index**	Optimal	1722	1.00		1.00		1.00	
	Overweight	1388	1.19 (1.11–1.28)	<0.0001	1.24 (1.15–1.34)	<0.0001	1.22 (1.13–1.32)	<0.0001
	Obesity	215	1.76 (1.52–2.02)	<0.0001	1.80 (1.56–2.08)	<0.0001	1.73 (1.49–2.00)	<0.0001
**Smoking**	Non-smoker	1382	1.00		1.00		1.00	
	Ex-smoker	1237	1.03 (0.96–1.11)	0.42	1.05 (0.97–1.13)	0.24	1.00 (0.92–1.08)	0.93
	Smoker	706	1.40 (1.28–1.53)	<0.0001	1.41 (1.29–1.55)	<0.0001	1.37 (1.25–1.50)	<0.0001
**Depression**	No	2561	1.00		1.00		1.00	
	Yes	764	1.21 (1.11–1.31)	<0.0001	1.21 (1.11–1.31)	<0.0001	1.17 (1.07–1.27)	0.0003
**Dyslipidemia**	No	2758	1.00		1.00		1.00	
	Yes	567	1.22 (1.11–1.33)	<0.0001	1.24 (1.13–1.36)	<0.0001	1.17 (1.06–1.28)	0.001
**Gender**	Women	773	1.00		1.00		1.00	
	Men	2552	0.96 (0.88–1.04)	0.30	0.97 (0.89–1.05)	0.42	0.87 (0.79–0.95)	0.003
**Hypertension**	No	3016	1.00		1.00		1.00	
	Yes	309	1.23 (1.10–1.39)	0.0006	1.24 (1.10–1.40)	0.0004	1.14 (1.01–1.28)	0.04

Only predictive factors significantly associated with incident physical inactivity in the multi-adjusted model are shown in the table.

**Table 6**shows that the incidence of obesity (1813 cases among 10,267 volunteers during follow-up (mean/median 8.4/8.0 years for volunteers with incident cases, 19.7/20.0 years for volunteers without incident cases) or 8.8 cases per 1000 person-years) is independently predicted by 5 factors with multi-adjusted HRs varying from 1.20 to 1.90.

**Table 6:** Risk of obesity according to predictive factors at baseline.

		No. of cases	Unadjusted		Gender & age-adjusted		Multi-adjusted	
			HR (95% CI)	p	HR (95% CI)	p	HR (95% CI)	p
**Hypertension**	No	1550	1.00		1.00		1.00	
	Yes	263	2.00 (1.75–2.28)	<0.0001	2.03 (1.78–2.31)	<0.0001	1.90 (1.66–2.16)	<0.0001
**Smoking**	Non-smoker	631	1.00		1.00		1.00	
	Ex-smoker	779	1.54 (1.39–1.71)	<0.0001	1.55 (1.39–1.73)	<0.0001	1.53 (1.37–1.71)	<0.0001
	Smoker	403	1.60 (1.41–1.81)	<0.0001	1.60 (1.41–1.82)	<0.0001	1.59 (1.40–1.81)	<0.0001
**Physical activity**	No	703	1.00		1.00		1.00	
	Yes	1110	0.64 (0.58–0.71)	<0.0001	0.63 (0.57–0.69)	<0.0001	0.67 (0.61–0.74)	<0.0001
**Dyslipidemia**	No	1464	1.00		1.00		1.00	
	Yes	349	1.36 (1.21–1.53)	<0.0001	1.37 (1.21–1.53)	<0.0001	1.23 (1.09–1.38)	0.001
**Depression**	No	1333	1.00		1.00		1.00	
	Yes	480	1.28 (1.15–1.42)	<0.0001	1.29 (1.16–1.43)	<0.0001	1.20 (1.07–1.33)	0.001

Only predictive factors significantly associated with incident obesity in the multi-adjusted model are shown in the table.

The incidence of hypertension (3950 cases among 9918 volunteers during follow-up (mean/median 9.9/10.0 years for volunteers with incident cases, 19.6/20.0 years for volunteers without incident cases) or 19.9 cases per 1000 person-years) is independently predicted by 8 factors with multi-adjusted HRs ranging from 1.09 to 2.78 (**Table 7**).

**Table 7:** Risk of hypertension according to predictive factors at baseline.

		No. of cases	Unadjusted		Gender & age-adjusted		Multi-adjusted	
			HR (95% CI)	p	HR (95% CI)	p	HR (95% CI)	p
**Body mass index**	Optimal	1732	1.00		1.00		1.00	
	Overweight	1889	1.72 (1.61–1.84)	<0.0001	1.73 (1.62–1.86)	<0.0001	1.69 (1.57–1.81)	<0.0001
	Obesity	329	2.94 (2.61–3.31)	<0.0001	2.94 (2.61–3.31)	<0.0001	2.78 (2.46–3.14)	<0.0001
**Age (y)**	39–45	1302	1.00		1.00		1.00	
	46–49	1307	1.23 (1.14–1.33)	<0.0001	1.20 (1.11–1.31)	<0.0001	1.18 (1.08–1.28)	<0.0001
	50–54	1341	1.40 (1.29–1.51)	<0.0001	1.37 (1.26–1.48)	<0.0001	1.32 (1.21–1.42)	<0.0001
**Diabetes**	No	3880	1.00		1.00		1.00	
	Yes	70	1.61 (1.26–2.02)	0.0002	1.53 (1.20–1.93)	0.0009	1.29 (1.01–1.63)	0.04
**Dyslipidemia**	No	3224	1.00		1.00		1.00	
	Yes	726	1.41 (1.30–1.53)	<0.0001	1.36 (1.25–1.48)	<0.0001	1.27 (1.17–1.37)	<0.0001
**Parental CVD**	No	3418	1.00		1.00		1.00	
	Yes	532	1.24 (1.13–1.36)	<0.0001	1.26 (1.15–1.38)	<0.0001	1.21 (1.10–1.33)	<0.0001
**Sleep disorder**	No	2843	1.00		1.00		1.00	
	Yes	1107	1.18 (1.10–1.26)	<0.0001	1.20 (1.12–1.29)	<0.0001	1.16 (1.08–1.24)	<0.0001
**Depression**	No	2991	1.00		1.00		1.00	
	Yes	959	1.19 (1.11–1.28)	<0.0001	1.19 (1.11–1.28)	<0.0001	1.14 (1.06–1.23)	0.0007
**Physical activity**	No	1308	1.00		1.00		1.00	
	Yes	2642	0.83 (0.78–0.89)	<0.0001	0.83 (0.78–0.89)	<0.0001	0.91 (0.85–0.98)	0.01

Only predictive factors significantly associated with incident hypertension in the multi-adjusted model are shown in the table.

Nine factors independently predict the incidence of dyslipidemia (4426 cases among 9257 volunteers during follow-up (mean/median 8.1/8.0 years for volunteers with incident cases, 19.5/20.0 years for volunteers without incident cases) or 23.9 cases per 1000 person-years) with multi-adjusted HRs varying from 1.09 to 1.51 (**Table 8**).

**Table 8:** Risk of dyslipidemia according to predictive factors at baseline.

		No. of cases	Unadjusted		Gender & age-adjusted		Multi-adjusted	
			HR (95% CI)	p	HR (95% CI)	p	HR (95% CI)	p
**Hypertension**	No	3921	1.00		1.00		1.00	
	Yes	505	1.66 (1.51–1.82)	<0.0001	1.63 (1.48–1.78)	<0.0001	1.51 (1.37–1.65)	<0.0001
**Body mass index**	Optimal	2257	1.00		1.00		1.00	
	Overweight	1871	1.35 (1.27–1.43)	<0.0001	1.32 (1.24–1.41)	<0.0001	1.27 (1.19–1.36)	<0.0001
	Obesity	298	1.60 (1.42–1.81)	<0.0001	1.57 (1.38–1.77)	<0.0001	1.39 (1.23–1.58)	<0.0001
**Diabetes**	No	4360	1.00		1.00		1.00	
	Yes	66	1.60 (1.24–2.02)	0.0004	1.53 (1.19–1.94)	0.001	1.38 (1.07–1.75)	0.01
**Age (y)**	39–45	1459	1.00		1.00		1.00	
	46–49	1462	1.20 (1.11–1.29)	<0.0001	1.17 (1.09–1.27)	<0.0001	1.14 (1.06–1.23)	0.0006
	50–54	1505	1.31 (1.22–1.41)	<0.0001	1.28 (1.19–1.38)	<0.0001	1.22 (1.13–1.32)	<0.0001
**Smoking**	Non-smoker	1896	1.00		1.00		1.00	
	Ex-smoker	1657	1.16 (1.08–1.24)	<0.0001	1.13 (1.06–1.21)	0.0004	1.08 (1.01–1.16)	0.03
	Smoker	873	1.22 (1.13–1.32)	<0.0001	1.21 (1.12–1.31)	<0.0001	1.18 (1.09–1.28)	<0.0001
**Depression**	No	3345	1.00		1.00		1.00	
	Yes	1081	1.18 (1.10–1.27)	<0.0001	1.19 (1.11–1.27)	<0.0001	1.13 (1.05–1.21)	0.0008
**Sleep disorder**	No	3190	1.00		1.00		1.00	
	Yes	1236	1.13 (1.06–1.21)	0.0002	1.16 (1.08–1.24)	<0.0001	1.11 (1.04–1.19)	0.002
**Parental CVD**	No	3872	1.00		1.00		1.00	
	Yes	554	1.14 (1.04–1.24)	0.005	1.15 (1.05–1.25)	0.003	1.10 (1.01–1.21)	0.03
**Physical activity**	No	1458	1.00		1.00		1.00	
	Yes	2968	0.86 (0.81–0.91)	<0.0001	0.86 (0.80–0.91)	<0.0001	0.91 (0.86–0.98)	0.007

Only predictive factors significantly associated with incident dyslipidemia in the multi-adjusted model are shown in the table.

**Table 9**shows that 7 factors independently predict the incidence of diabetes (1138 cases among 10,578 volunteers during follow-up (mean/median 10.9/11.0 years for volunteers with incident cases, 19.7/20.0 years for volunteers without incident cases) or 5.4 cases per 1000 person-years). with multi-adjusted HRs ranging from 1.15 to 6.12.

**Table 9:** Risk of diabetes according to predictive factors at baseline.

		No. of cases	Unadjusted		Gender & age-adjusted		Multi-adjusted	
			HR (95% CI)	p	HR (95% CI)	p	HR (95% CI)	p
**Body mass index**	Optimal	319	1.00		1.00		1.00	
	Overweight	594	2.59 (2.27–2.98)	<0.0001	2.46 (2.13–2.84)	<0.0001	2.23 (1.93–2.58)	<0.0001
	Obesity	225	8.01 (6.75–9.50)	<0.0001	7.69 (6.46–9.14)	<0.0001	6.12 (5.10–7.33)	<0.0001
**Hypertension**	No	910	1.00		1.00		1.00	
	Yes	228	2.69 (2.32–3.10)	<0.0001	2.62 (2.26–3.02)	<0.0001	1.75 (1.50–2.04)	<0.0001
**Dyslipidemia**	No	824	1.00		1.00		1.00	
	Yes	314	2.19 (1.92–2.49)	<0.0001	2.03 (1.78–2.32)	<0.0001	1.66 (1.45–1.89)	<0.0001
**Smoking**	Non-smoker	373	1.00		1.00		1.00	
	Ex-smoker	489	1.58 (1.38–1.81)	<0.0001	1.44 (1.25–1.65)	<0.0001	1.18 (1.02–1.36)	0.02
	Smoker	276	1.85 (1.58–2.16)	<0.0001	1.74 (1.49–2.04)	<0.0001	1.57 (1.34–1.84)	<0.0001
**Depression**	No	823	1.00		1.00		1.00	
	Yes	315	1.36 (1.19–1.55)	<0.0001	1.37 (1.20–1.56)	<0.0001	1.25 (1.09–1.43)	0.001
**Parental CVD**	No	964	1.00		1.00		1.00	
	Yes	174	1.32 (1.12–1.55)	0.001	1.35 (1.14–1.58)	0.0005	1.21 (1.02–1.42)	0.02
**Physical activity**	No	446	1.00		1.00		1.00	
	Yes	692	0.67 (0.60–0.76)	<0.0001	0.66 (0.58–0.74)	<0.0001	0.85 (0.75–0.96)	0.01

Only predictive factors significantly associated with incident diabetes in the multi-adjusted model are shown in the table.

The incidence of sleep disorder (5226 cases among 8815 volunteers during follow-up (mean/median 5.7/4.0 years for volunteers with incident cases, 19.7/20.0 years for volunteers without incident cases) or 29.6 cases per 1000 person-years) is independently predicted by 5 factors with multi-adjusted HRs ranging from 1.13 to 1.88 (**Table 10**).

**Table 10:** Risk of sleep disorder according to predictive factors at baseline.

		No. of cases	Unadjusted		Gender & age-adjusted		Multi-adjusted	
			HR (95% CI)	p	HR (95% CI)	p	HR (95% CI)	p
**Depression**	No	3949	1.00		1.00		1.00	
	Yes	1277	1.86 (1.75–1.98)	<0.0001	1.88 (1.77–2.00)	<0.0001	1.88 (1.76–2.00)	<0.0001
**Gender**	Women	1586	1.00		1.00		1.00	
	Men	3640	0.57 (0.54–0.61)	<0.0001	0.58 (0.55–0.62)	<0.0001	0.56 (0.52–0.60)	<0.0001
**Age (y)**	39–45	1723	1.00		1.00		1.00	
	46–49	1726	0.90 (0.84–0.96)	0.001	1.03 (0.96–1.10)	0.41	1.03 (0.96–1.10)	0.43
	50–54	1777	0.78 (0.73–0.83)	<0.0001	0.89 (0.83–0.95)	0.0009	0.88 (0.82–0.94)	0.0002
**Parental CVD**	No	4554	1.00		1.00		1.00	
	Yes	672	1.17 (1.08–1.27)	0.0002	1.15 (1.06–1.25)	0.0008	1.14 (1.05–1.24)	0.002
**Smoking**	Non-smoker	2361	1.00		1.00		1.00	
	Ex-smoker	1886	0.92 (0.86–0.97)	0.005	1.06 (0.99–1.13)	0.08	1.04 (0.97–1.11)	0.25
	Smoker	979	1.04 (0.97–1.12)	0.29	1.15 (1.07–1.24)	0.0003	1.13 (1.05–1.22)	0.002

Only predictive factors significantly associated with incident sleep disorder in the multi-adjusted model are shown in the table.

Four factors independently predict the incidence of depression (2519 cases among 8314 volunteers during follow-up (mean/median 6.8/6.0 years for volunteers with incident cases, 19.8/20.0 years for volunteers without incident cases) or 15.1 cases per 1000 person-years) with multi-adjusted HRs varying from 1.11 to 1.78 (**Table 11**).

**Table 11:** Risk of depression according to predictive factors at baseline.

		No. of cases	Unadjusted		Gender & age-adjusted		Multi-adjusted	
			HR (95% CI)	p	HR (95% CI)	p	HR (95% CI)	p
**Sleep disorder**	No	1778	1.00		1.00		1.00	
	Yes	741	1.80 (1.65–1.96)	<0.0001	1.78 (1.63–1.94)	<0.0001	1.77 (1.62–1.93)	<0.0001
**Age (y)**	39–45	831	1.00		1.00		1.00	
	46–49	832	0.91 (0.83–0.99)	0.04	0.93 (0.85–1.03)	0.16	0.92 (0.83–1.01)	0.08
	50–54	856	0.77 (0.70–0.84)	<0.0001	0.79 (0.71–0.87)	<0.0001	0.78 (0.70–0.86)	<0.0001
**Smoking**	Non-smoker	1081	1.00		1.00		1.00	
	Ex-smoker	918	1.01 (0.92–1.10)	0.87	1.06 (0.96–1.16)	0.23	1.02 (0.93–1.12)	0.62
	Smoker	520	1.22 (1.10–1.35)	0.0002	1.25 (1.13–1.39)	<0.0001	1.22 (1.10–1.36)	0.0003
**Parental CVD**	No	2175	1.00		1.00		1.00	
	Yes	344	1.18 (1.06–1.33)	0.004	1.17 (1.05–1.31)	0.007	1.14 (1.01–1.27)	0.03

Only predictive factors significantly associated with incident depression in the multi-adjusted model are shown in the table.

### Overview of the associations between the risk factors

**Table 12**summarizes multi-adjusted HRs for all the associations that have been observed between the risk factors. The number of associations is 47 at p<0.05, 37 at p<0.01, 28 at p<0.001 and 22 at p<0.0001. The number of factors associated with a given factor varies from 1 to 9 at p<0.05 (from 0 to 6 at p<0.0001) with a number of predictive or predicted factors ranging respectively from 0 to 9 and from 0 to 7 at p<0.05 (from 0 to 5 and from 0 to 4 at p<0.0001) as reported in **Table 13**.

**Table 12:** Risk of incident CVD factors according to the same factors at baseline—summary of multi-adjusted associations.

Predictive factors		Incident factors								
		Diabetes	Smoking	Hypertension	Obesity	Non-moderate alcohol consumption	Sleep disorder	Dyslipidemia	Physical inactivity	Depression
**Diabetes**	No	-	NS	1.00	NS	NS	NS	1.00	NS	NS
	Yes			1.29 (1.01–1.63)				1.38 (1.07–1.75)		
**Gender**	Women	NS	NS	NS	NS	NS	1.00	NS	1.00	NS
	Men						0.56 (0.52–0.60)		0.87 (0.79–0.95)	
**Smoking**	Non-smoker	1.00		NS	1.00	1.00	1.00	1.00	1.00	1.00
	Ex-smoker	1.18 (1.02–1.36)	-		1.53 (1.37–1.71)	1.13 (1.03–1.24)	1.04 (0.97–1.11)	1.08 (1.01–1.16)	1.00 (0.92–1.08)	1.02 (0.93–1.12)
	Smoker	1.57 (1.34–1.84)			1.59 (1.40–1.81)	1.14 (1.02–1.27)	1.13 (1.05–1.22)	1.18 (1.09–1.28)	1.37 (1.25–1.50)	1.22 (1.10–1.36)
**Age (y)**	39–45	NS	NS	1.00	NS	NS	1.00	1.00	NS	1.00
	46–49			1.18 (1.08–1.28)			1.03 (0.96–1.10)	1.14 (1.06–1.23)		0.92 (0.83–1.01)
	50–54			1.32 (1.21–1.42)			0.88 (0.82–0.94)	1.22 (1.13–1.32)		0.78 (0.70–0.86)
**Hypertension**	No	1.00	NS	-	1.00	NS	NS	1.00	1.00	NS
	Yes	1.75 (1.50–2.04)			1.90 (1.66–2.16)			1.51 (1.37–1.65)	1.14 (1.01–1.28)	
**Body mass index**	Optimal	1.00	1.00	1.00		NS	NS	1.00	1.00	NS
	Overweight	2.23 (1.93–2.58)	1.29 (1.12–1.47)	1.69 (1.57–1.81)	-			1.27 (1.19–1.36)	1.22 (1.13–1.32)	
	Obesity	6.12 (5.10–7.33)	1.27 (0.97–1.64)	2.78 (2.46–3.14)				1.39 (1.23–1.58)	1.73 (1.49–2.00)	
**Parental CVD**	No	1.00	NS	1.00	NS	NS	1.00	1.00	NS	1.00
	Yes	1.21 (1.02–1.42)		1.21 (1.10–1.33)			1.14 (1.05–1.24)	1.10 (1.01–1.21)		1.14 (1.01–1.27)
**Alcohol consumption**	Non-drinker	NS	NS	NS	NS	-	NS	NS	NS	NS
	Light drinker									
	Moderate drinker									
	Heavy drinker									
**Sleep disorder**	No	NS	NS	1.00	NS	NS	-	1.00	NS	1.00
	Yes			1.16 (1.08–1.24)				1.11 (1.04–1.19)		1.77 (1.62–1.93)
**Dyslipidemia**	No	1.00	NS	1.00	1.00	NS	NS	-	1.00	NS
	Yes	1.66 (1.45–1.89)		1.27 (1.17–1.37)	1.23 (1.09–1.38)				1.17 (1.06–1.28)	
**Physical activity**	No	1.00	NS	1.00	1.00	NS	NS	1.00	-	NS
	Yes	0.85 (0.75–0.96)		0.91 (0.85–0.98)	0.67 (0.61–0.74)			0.91 (0.86–0.98)		
**Depression**	No	1.00	1.00	1.00	1.00	NS	1.00	1.00	1.00	-
	Yes	1.25 (1.09–1.43)	1.18 (1.02–1.37)	1.14 (1.06–1.23)	1.20 (1.07–1.33)		1.88 (1.76–2.00)	1.13 (1.05–1.21)	1.17 (1.07–1.27)	

HRs (95% CI). White background: p<0.05, light gray: p<0.01, middle gray: p<0.001, dark gray: p<0.0001, NS: non-significant.

**Table 13:** Summary of the numbers of predictive and predicted factors and of the total number of factors independently associated with each CVD risk factor according to the significance threshold.

		Significance threshold	Number of predictive factors	Number of predicted factors	Total number of associated factors
**Non-modifiable factors**	**Gender**	<0.05	0	2	2
		<0.01	0	2	2
		<0.001	0	1	1
		<0.0001	0	1	1
	**Age**	<0.05	0	4	4
		<0.01	0	4	4
		<0.001	0	4	4
		<0.0001	0	3	3
	**Parental CVD**	<0.05	0	5	5
		<0.01	0	2	2
		<0.001	0	1	1
		<0.0001	0	1	1
**Behavioural factors**	**Alcohol consumption**	<0.05	1	0	1
		<0.01	0	0	0
		<0.001	0	0	0
		<0.0001	0	0	0
	**Smoking**	<0.05	2	7	7
		<0.01	1	6	6
		<0.001	1	5	5
		<0.0001	0	4	4
	**Physical activity**	<0.05	6	4	7
		<0.01	5	2	5
		<0.001	3	1	4
		<0.0001	2	1	2
**Upstream clinical factors**	**Obesity**	<0.05	5	5	6
		<0.01	5	5	6
		<0.001	3	5	5
		<0.0001	3	4	5
	**Sleep disorder**	<0.05	5	3	7
		<0.01	5	3	7
		<0.001	3	2	4
		<0.0001	2	2	3
	**Depression**	<0.05	4	7	9
		<0.01	3	6	8
		<0.001	3	4	6
		<0.0001	2	1	2
**Downstream clinical factors**	**Hypertension**	<0.05	8	4	8
		<0.01	6	3	7
		<0.001	6	3	7
		<0.0001	5	3	6
	**Dyslipidemia**	<0.05	9	4	9
		<0.01	7	4	8
		<0.001	5	2	6
		<0.0001	4	2	5
	**Diabetes**	<0.05	7	2	7
		<0.01	5	0	5
		<0.001	4	0	4
		<0.0001	4	0	4

For each factor, the number of predictive factors refers to the number of factors predicting this factor in the corresponding regression while the number of predicted factors designates the number of factors predicted by this factor in all the regressions.

## Discussion

The present study systematically reports prospective associations between the main behavioural or clinical CVD risk factors in a cohort of middle-aged volunteers free of CVD at baseline and followed over 20 years. The 12 factors retained for the analyses are all independently associated with the risk of CVD events in a dose-dependent manner for non-binary factors and in agreement with the literature [3, 4, 7, 8, 10, 14, 36–42]. Beside their independent associations with CVD events, our results show that these risk factors form an extensive network of relationships with each other. The most straightforward observation that can be made from the overall view of this network is that the number of factors associated with a given factor, as well as the number of factors predicting a given factor or predicted by it, are quite variable, suggesting that all the factors do not have an equivalent position within the network. A conceptual framework can be tentatively proposed in which four types of factors are identified on the basis of their nature and of the number of relationships they formed with each other (**Fig 1**). Non-modifiable factors (gender, age, parental history of CVD) obviously only predict and are not predicted by other factors. Behavioural factors (non-moderate drinking, smoking, physical inactivity) form very few associations with each other, they predict several clinical factors and are predicted by a small number of non-modifiable or clinical factors. Upstream clinical factors (obesity, sleep disorder, depression) form a few associations with each other, they predict many downstream clinical factors and are predicted by many non-modifiable or behavioural factors. Downstream clinical factors (hypertension, dyslipidemia, diabetes) form many associations with each other, they predict very few factors but are predicted by a large number of non-modifiable, behavioural factors or upstream clinical factors.

In theory, the extent of this network of relationships between the risk factors underlines the necessity of considering all the factors together to evaluate CVD risk. Approaches such as structural analyses should allow to determine the relative contribution of each factor to the overall CVD risk and to explore what type of relationships (additive, synergistic) exists between the different factors [21]. An adjacent question would be to disentangle how the contribution of each factor divides up between its independent effect and the effect mediated by the relationships with the other factors, or in other words, how the risk predicted by a given factor varied in relation to the burden of interacting factors [1]. However, from a practical viewpoint, it has been demonstrated that 75–90% of coronary heart disease incidence in most populations is explained by only a few key risk factors, mainly the downstream clinical factors funnelling the effects of the other factors within the network (hypertension, dyslipidemia, diabetes) [43]. It has also been shown that adding new factors besides the key ones to existing algorithms and scores only marginally improves risk prediction [25]. Thus, despite its potential theoretical interest, taking into account the interaction network between CVD risk factors will probably not add much to the identification of individuals at risk.

In contrast, integrating this network in the design and implementation of primary or secondary preventive strategies aiming to lower CVD risk, whose efficiency has remained very disappointing [26, 27], may have some utility. One reason of the lack of success of these strategies lies in the intensity of the interventions that is usually insufficient to significantly reduce exposure to the risk factors. The global view of the interaction network between these risk factors suggests another reason: not taking into account the relationships between the factors can lead to partial or contradictory interventions if the simultaneous presence of these factors is susceptible to appreciably influence the reduction of the exposure to any one of them. For example, when it is necessary to manage diabetes in obese patients who are also smokers and depressed. Generally, the relationships formed by downstream clinical factors should be given a particular attention considering that they are predicted by a large number of other factors.

The present study has several limitations. A first one concerns the very limited set of CVD risk factors that have been tested among all those which are available [44]. A second limitation is that the diagnosis of behaviours, clinical phenotypes and CVD outcomes could suffer from significant imprecisions as they were self-reported, even though the fact that all the risk factors predict CVD events as expected and that many associations between these factors are also in agreement with the literature is reassuring. A third limitation concerns the potential generalization of the findings that may be limited due to the specific characteristics of the present cohort. In addition to the unusual sex ratio and rather narrow age range, the selection of volunteers working in a French public company necessarily leads to a healthy cohort as shown by the low prevalence/incidence of CVD events and risk factors [45], a bias further accentuated by the exclusion of workers with missing values. It is therefore difficult to appreciate the applicability of the findings to other contexts until similar analyses are performed in cohorts with different characteristics. A fourth limitation concerns the interpretation of the associations identified by proportional hazard regression. Some of them such as the prediction of hypertension or diabetes by body mass index probably correspond to cause and effect relationships as demonstrated by intervention studies [46, 47]. But other associations such as those between hypertension, dyslipidemia and diabetes are less likely to be causal and would rather reflect an underlying common pathological process that develops over the long-term. This process would increase the risk of occurrence of these phenotypes at different rates, resulting in the apparent prediction of some phenotypes by those already present at baseline. Thus, the number of causal relationships between the risk factors is probably significantly less than the number of associations reported in the results. A fifth limitation is related to the potential specificities of predictive and predicted risk factors which have not been assessed in this study. Although a given risk factor is a well-defined behavioural or clinical phenotype, it presents some differences when acting as predictive or predicted factor. As predictive factor, it reflects the prevalence of the phenotype at the beginning of the follow-up while as predicted factor it reflects the incidence of the phenotype during the follow-up. In the second case, it is the frequency of appearance of the phenotype over time while in the first case it is the result of the past incidence of the phenotype accumulated over some period of time during which the volunteers were exposed to the phenotype before the beginning of the follow-up. Generally, the risk associated with a given predicted/incident factor will not be identical to that associated with the corresponding predictive/prevalent factor because this is likely to depend on the duration of the exposure to the factor.

## Conclusions

The present study assessed in a single cohort all prospective associations between the main behavioural and clinical CVD risk factors. It provides a global view of the extensive network of relationships formed by these factors in parallel with their independent associations with CVD risk, highlighting the inadequacy of considering a factor or even a few without taking into account the interactions with the other factors. The extent of this network is probably underestimated and not sufficiently taken into account in the preventive strategies aiming to lower CVD risk, explaining perhaps in part their lack of efficiency.

## Supporting Information

S1 Fig Number of volunteers at risk during follow-up. (DOCX)

S2 Fig Stratified Kaplan-Meier curves. (DOCX)

S3 Fig Kaplan-Meier curve for death. (DOCX)

S4 Fig Overall pattern of missing data. (DOCX)

S1 Table Multi-adjusted Cox regressions without excluding loss to follow-up volunteers. (DOCX)

S2 Table Comparison of baseline characteristics between excluded and included volunteers. (DOCX)

S3 Table Weighted multi-adjusted Cox regressions. (DOCX)

S4 Table Gender-age adjusted Cox regressions. (DOCX)

S5 Table Multi-adjusted Cox regressions with time-independent or time-dependent risk factors. (DOCX)

## References

[1] Kannel WB, D’Agostino RB, Sullivan L, Wilson PW (2004) Concept and usefulness of cardiovascular risk profiles. Am Heart J 148: 16–26. 15215787 10.1016/j.ahj.2003.10.022

[2] Payne RA (2012) Cardiovascular risk. Br J Clin Pharmacol 74: 396–410. 10.1111/j.1365-2125.2012.04219.x 22348281 PMC3477342

[3] Erhardt L (2009) Cigarette smoking: an undertreated risk factor for cardiovascular disease. Atherosclerosis 205: 23–32. 10.1016/j.atherosclerosis.2009.01.007 19217623

[4] Ronksley PE, Brien SE, Turner BJ, Mukamal KJ, Ghali WA (2011) Association of alcohol consumption with selected cardiovascular disease outcomes: a systematic review and meta-analysis. BMJ 342: d671 10.1136/bmj.d671 21343207 PMC3043109

[5] Li J, Siegrist J (2012) Physical activity and risk of cardiovascular disease—a meta-analysis of prospective cohort studies. Int J Environ Res Public Health 9: 391–407. 10.3390/ijerph9020391 22470299 PMC3315253

[6] Kannel WB (2002) The Framingham Study: historical insight on the impact of cardiovascular risk factors in men versus women. J Gend Specif Med 5: 27–37. 11974672

[7] Tuomilehto J (2004) Impact of age on cardiovascular risk: implications for cardiovascular disease management. Atheroscler Suppl 5: 9–17. 15121030 10.1016/j.atherosclerosissup.2004.03.006

[8] Kashani M, Eliasson A, Vernalis M, Costa L, Terhaar M (2013) Improving assessment of cardiovascular disease risk by using family history: an integrative literature review. J Cardiovasc Nurs 28: E18–27. 10.1097/JCN.0b013e318294b206 23782863

[9] Guo X, Zhang X, Guo L, Li Z, Zheng L, Yu S et al (2013) Association between pre-hypertension and cardiovascular outcomes: a systematic review and meta-analysis of prospective studies. Curr Hypertens Rep 15: 703–716. 24234576 10.1007/s11906-013-0403-y

[10] Wallace RB, Anderson RA (1987) Blood lipids, lipid-related measures, and the risk of atherosclerotic cardiovascular disease. Epidemiol Rev 9: 95–119. 3315721 10.1093/oxfordjournals.epirev.a036310

[11] Katzmarzyk PT, Reeder BA, Elliott S, Joffres MR, Pahwa P, Raine KD et al (2012) Body mass index and risk of cardiovascular disease, cancer and all-cause mortality. Can J Public Health 103: 147–151. 22530540 10.1007/BF03404221 PMC6974265

[12] Martin-Timon I, Sevillano-Collantes C, Segura-Galindo A, Del Canizo-Gomez FJ (2014) Type 2 diabetes and cardiovascular disease: Have all risk factors the same strength? World J Diabetes 5: 444–470. 10.4239/wjd.v5.i4.444 25126392 PMC4127581

[13] Kivimaki M, Nyberg ST, Batty GD, Fransson EI, Heikkila K, Alfredsson L et al (2012) Job strain as a risk factor for coronary heart disease: a collaborative meta-analysis of individual participant data. Lancet 380: 1491–1497. 10.1016/S0140-6736(12)60994-5 22981903 PMC3486012

[14] Cappuccio FP, Cooper D, D’Elia L, Strazzullo P, Miller MA (2011) Sleep duration predicts cardiovascular outcomes: a systematic review and meta-analysis of prospective studies. Eur Heart J 32: 1484–1492. 10.1093/eurheartj/ehr007 21300732

[15] Wang X, Ouyang Y, Wang Z, Zhao G, Liu L, Bi Y (2013) Obstructive sleep apnea and risk of cardiovascular disease and all-cause mortality: a meta-analysis of prospective cohort studies. Int J Cardiol 169: 207–214. 10.1016/j.ijcard.2013.08.088 24161531

[16] Van der Kooy K, van Hout H, Marwijk H, Marten H, Stehouwer C, Beekman A (2007) Depression and the risk for cardiovascular diseases: systematic review and meta analysis. Int J Geriatr Psychiatry 22: 613–626. 17236251 10.1002/gps.1723

[17] Anthony D, George P, Eaton CB (2014) Cardiac risk factors: biomarkers and genetic tests to determine cardiovascular risk. FP Essent 421: 11–15. 24936714

[18] Angelakopoulou A, Shah T, Sofat R, Shah S, Berry DJ, Cooper J et al (2012) Comparative analysis of genome-wide association studies signals for lipids, diabetes, and coronary heart disease: Cardiovascular Biomarker Genetics Collaboration. Eur Heart J 33: 393–407. 10.1093/eurheartj/ehr225 21804106 PMC3270041

[19] Castelli WP (1983) Cardiovascular disease and multifactorial risk: challenge of the 1980s. Am Heart J 106: 1191–1200. 6637784 10.1016/0002-8703(83)90174-6

[20] Siontis GC, Tzoulaki I, Siontis KC, Ioannidis JP (2012) Comparisons of established risk prediction models for cardiovascular disease: systematic review. BMJ 344: e3318 10.1136/bmj.e3318 22628003

[21] Murray CJ, Ezzati M, Lopez AD, Rodgers A, Vander Hoorn S (2003) Comparative quantification of health risks conceptual framework and methodological issues. Popul Health Metr 1: 1 12780936 10.1186/1478-7954-1-1 PMC156894

[22] Yusuf S, Hawken S, Ounpuu S, Dans T, Avezum A, Lanas F et al (2004) Effect of potentially modifiable risk factors associated with myocardial infarction in 52 countries (the INTERHEART study): case-control study. Lancet 364: 937–952. 15364185 10.1016/S0140-6736(04)17018-9

[23] O’Donnell MJ, Xavier D, Liu L, Zhang H, Chin SL, Rao-Melacini P et al (2010) Risk factors for ischaemic and intracerebral haemorrhagic stroke in 22 countries (the INTERSTROKE study): a case-control study. Lancet 376: 112–123. 10.1016/S0140-6736(10)60834-3 20561675

[24] Ganna A, Ingelsson E (2015) 5 year mortality predictors in 498,103 UK Biobank participants: a prospective population-based study. Lancet 386: 533–540. 10.1016/S0140-6736(15)60175-1 26049253

[25] De Backer G, Graham I, Cooney MT (2012) Do novel biomarkers add to existing scores of total cardiovascular risk? Eur J Prev Cardiol 19: 14–17. 10.1177/2047487312448988 22801065

[26] Kotseva K, Wood D, De Bacquer D, De Backer G, Rydén L, Jennings C et al (2015) EUROASPIRE IV: A European Society of Cardiology survey on the lifestyle, risk factor and therapeutic management of coronary patients from 24 European countries. Eur J Prev Cardiol 10.1177/2047487315569401 25687109

[27] Willis A, Davies M, Yates T, Khunti K (2012) Primary prevention of cardiovascular disease using validated risk scores: a systematic review. J R Soc Med 105: 348–356. 10.1258/jrsm.2012.110193 22907552 PMC3423129

[28] Goldberg M, Leclerc A, Bonenfant S, Chastang JF, Schmaus A, Kaniewski N et al (2007) Cohort profile: the GAZEL Cohort Study. Int J Epidemiol 36: 32–39. 17101614 10.1093/ije/dyl247 PMC2258334

[29] Zins M, Leclerc A, Goldberg M (2009) The french GAZEL cohort study: 20 years of epidemiological research. Advances in Life Course Research 14: 135–146.

[30] Le Port A, Gueguen A, Kesse-Guyot E, Melchior M, Lemogne C, Nabi H et al (2012) Association between dietary patterns and depressive symptoms over time: a 10-year follow-up study of the GAZEL cohort. PLoS One 7: e51593 10.1371/journal.pone.0051593 23251585 PMC3520961

[31] Tamers SL, Okechukwu C, Bohl AA, Gueguen A, Goldberg M, Zins M (2014) The impact of stressful life events on excessive alcohol consumption in the French population: findings from the GAZEL cohort study. PLoS One 9: e87653 10.1371/journal.pone.0087653 24475318 PMC3903768

[32] Metzger MH, Goldberg M, Chastang JF, Leclerc A, Zins M (2002) Factors associated with self-reporting of chronic health problems in the French GAZEL cohort. J Clin Epidemiol 55: 48–59. 11781122 10.1016/s0895-4356(01)00409-7

[33] Noordzij M, Leffondré K, van Stralen KJ, Zoccali C, Dekker FW, Jager KJ (2013) When do we need competing risks methods for survival analysis in nephrology. Nephrol Dial Transplant 28: 2670–2677. 10.1093/ndt/gft355 23975843

[34] Allison PD (2001) Missing data. Sage University Papers Series on Quantitative Applications in the Social Sciences 07–136.

[35] White IR, Carlin JB (2010) Bias and efficiency of multiple imputation compared with complete-case analysis for missing covariate values. Stat Med 29: 2920–2931. 10.1002/sim.3944 20842622

[36] Dong JY, Zhang YH, Qin LQ (2013) Obstructive sleep apnea and cardiovascular risk: meta-analysis of prospective cohort studies. Atherosclerosis 229: 489–495. 10.1016/j.atherosclerosis.2013.04.026 23684511

[37] Fiedorowicz JG (2014) Depression and cardiovascular disease: an update on how course of illness may influence risk. Curr Psychiatry Rep 16: 492 10.1007/s11920-014-0492-6 25163592 PMC4153989

[38] Li J, Loerbroks A, Angerer P (2013) Physical activity and risk of cardiovascular disease: what does the new epidemiological evidence show? Curr Opin Cardiol 28: 575–583. 10.1097/HCO.0b013e328364289c 23928923

[39] Lu Y, Hajifathalian K, Ezzati M, Woodward M, Rimm EB, Danaei G (2014) Metabolic mediators of the effects of body-mass index, overweight, and obesity on coronary heart disease and stroke: a pooled analysis of 97 prospective cohorts with 1.8 million participants. Lancet 383: 970–983. 10.1016/S0140-6736(13)61836-X 24269108 PMC3959199

[40] Peters SA, Huxley RR, Woodward M (2013) Comparison of the sex-specific associations between systolic blood pressure and the risk of cardiovascular disease: a systematic review and meta-analysis of 124 cohort studies, including 1.2 million individuals. Stroke 44: 2394–2401. 10.1161/STROKEAHA.113.001624 23821229

[41] Peters SA, Huxley RR, Woodward M (2014) Diabetes as a risk factor for stroke in women compared with men: a systematic review and meta-analysis of 64 cohorts, including 775,385 individuals and 12,539 strokes. Lancet 383: 1973–1980. 10.1016/S0140-6736(14)60040-4 24613026

[42] Price JF, Fowkes FG (1997) Risk factors and the sex differential in coronary artery disease. Epidemiology 8: 584–591. 9270963 10.1097/00001648-199709000-00018

[43] Kannel WB, Vasan RS (2009) Adverse consequences of the 50% misconception. Am J Cardiol 103: 426–427. 10.1016/j.amjcard.2008.09.098 19166702 PMC3753109

[44] Hopkins PN, Williams RR (1986) Identification and relative weight of cardiovascular risk factors. Cardiol Clin 4: 3–31. 3518932

[45] Go AS, Mozaffarian D, Roger VL, Benjamin EJ, Berry JD, Blaha MJ et al (2014) Heart disease and stroke statistics—2014 update: a report from the American Heart Association. Circulation 129: e28–e292. 10.1161/01.cir.0000441139.02102.80 24352519 PMC5408159

[46] Geleijnse JM, Kok FJ, Grobbee DE (2004) Impact of dietary and lifestyle factors on the prevalence of hypertension in Western populations. Eur J Public Health 14: 235–239. 15369026 10.1093/eurpub/14.3.235

[47] Merlotti C, Morabito A, Pontiroli AE (2014) Prevention of type 2 diabetes; a systematic review and meta-analysis of different intervention strategies. Diabetes Obes Metab 16: 719–727. 10.1111/dom.12270 24476122

