# Supplementary figures and images for: A Global View of the Relationships between the Main Behavioural and Clinical Cardiovascular Risk Factors in the GAZEL Prospective Cohort
Source: PLoS One. 2016 Sep 6;11(9):e0162386. doi: 10.1371/journal.pone.0162386 (PMC5012694; doi:10.1371/journal.pone.0162386)

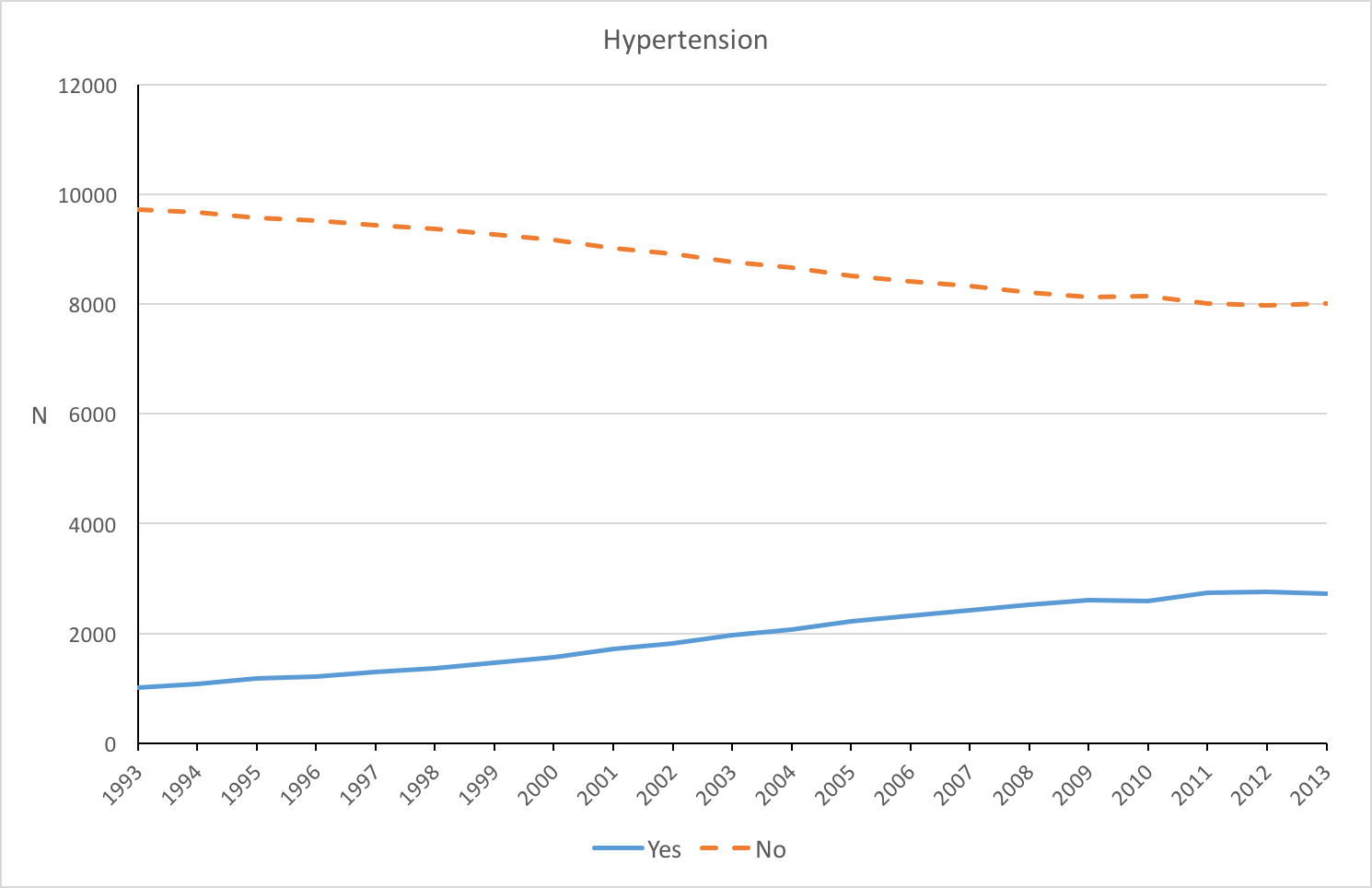


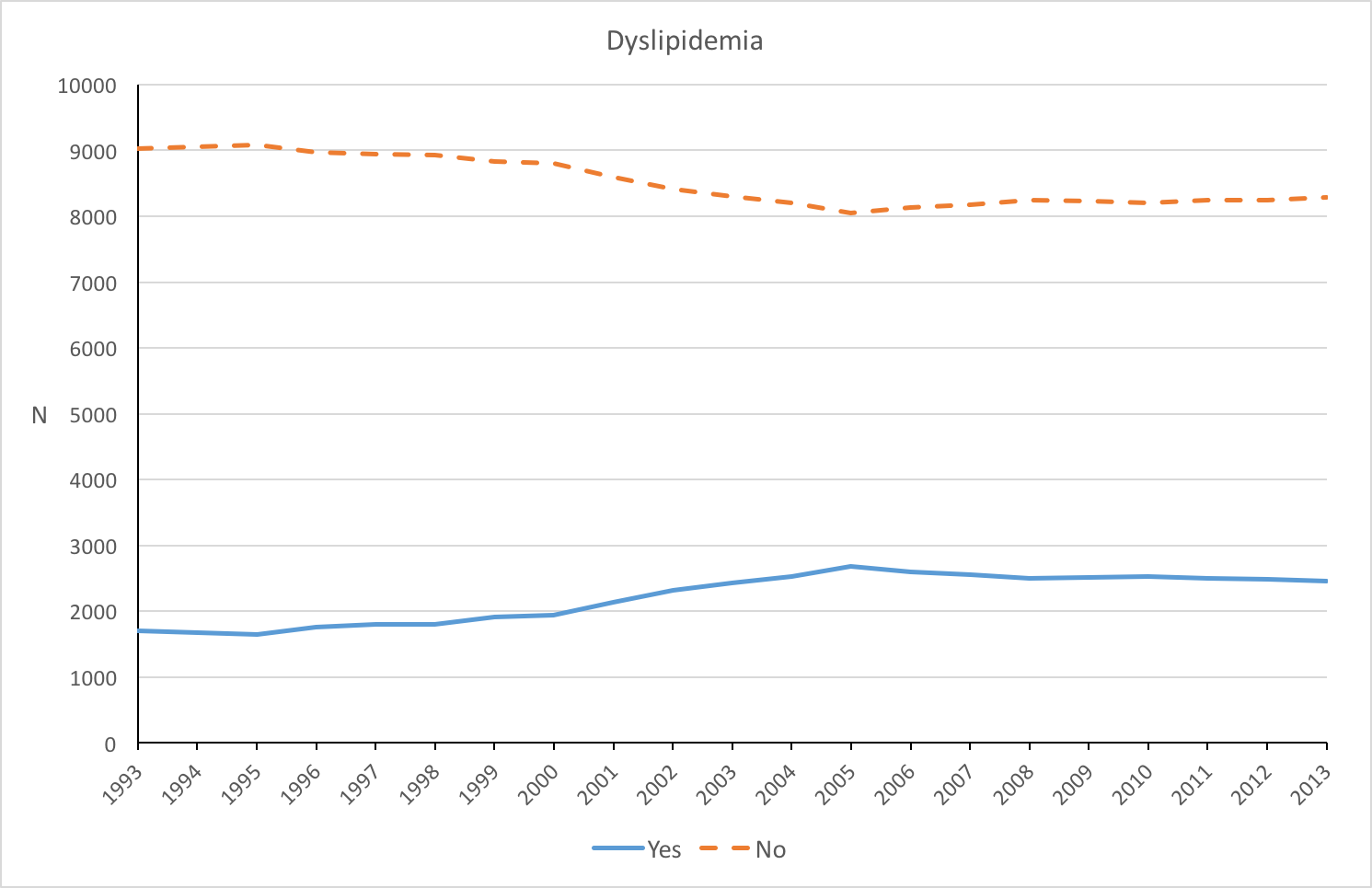


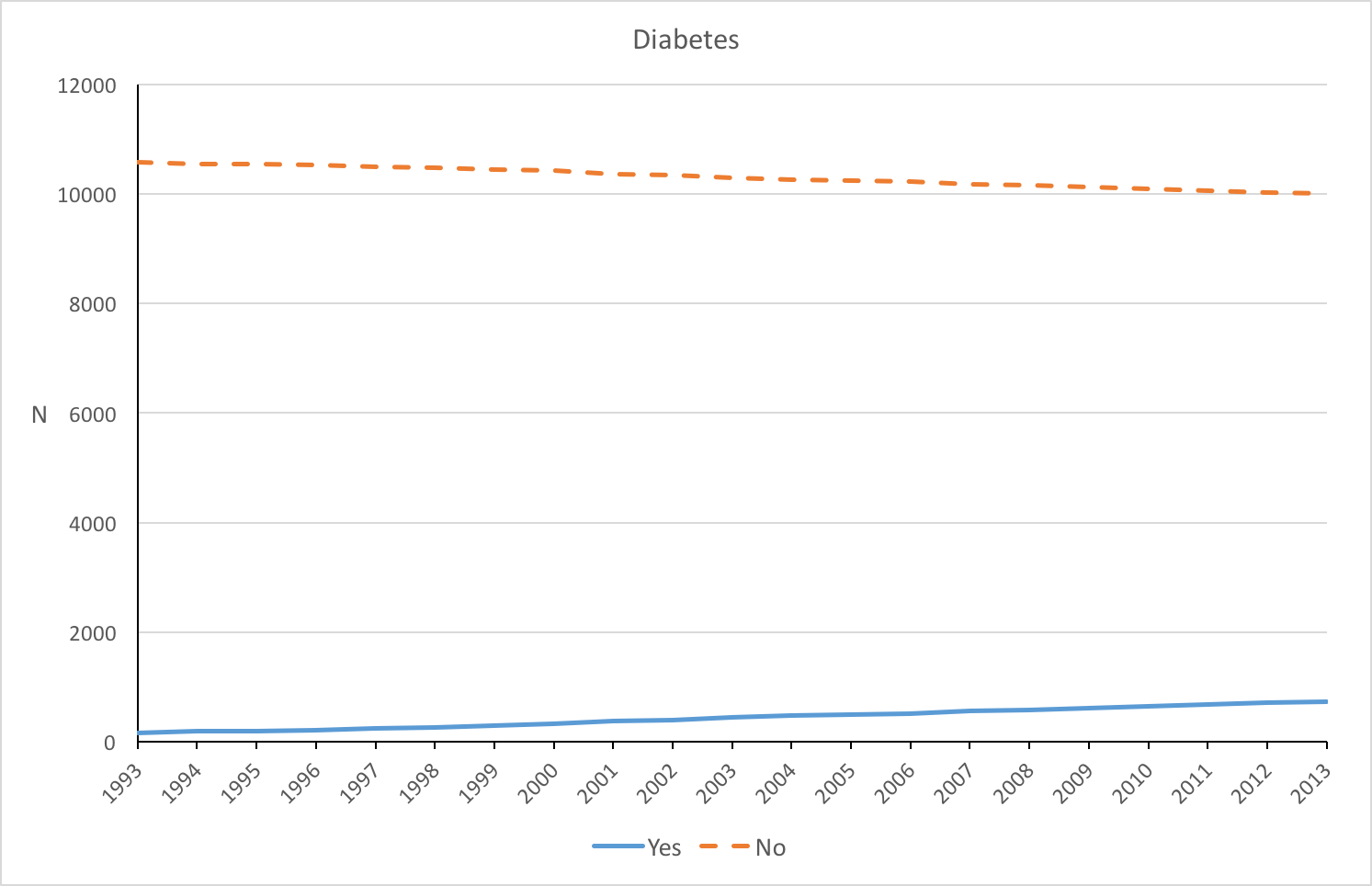


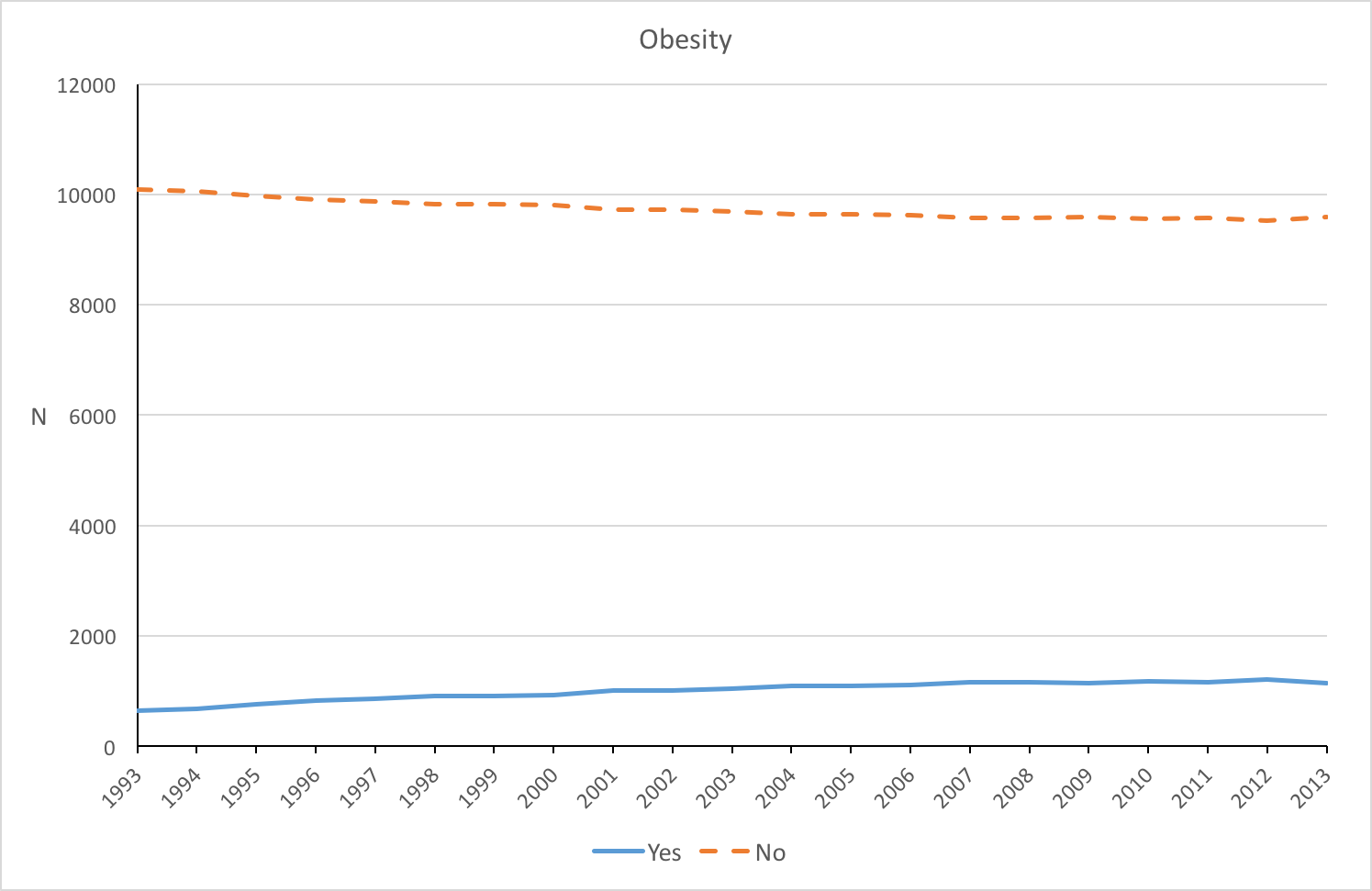


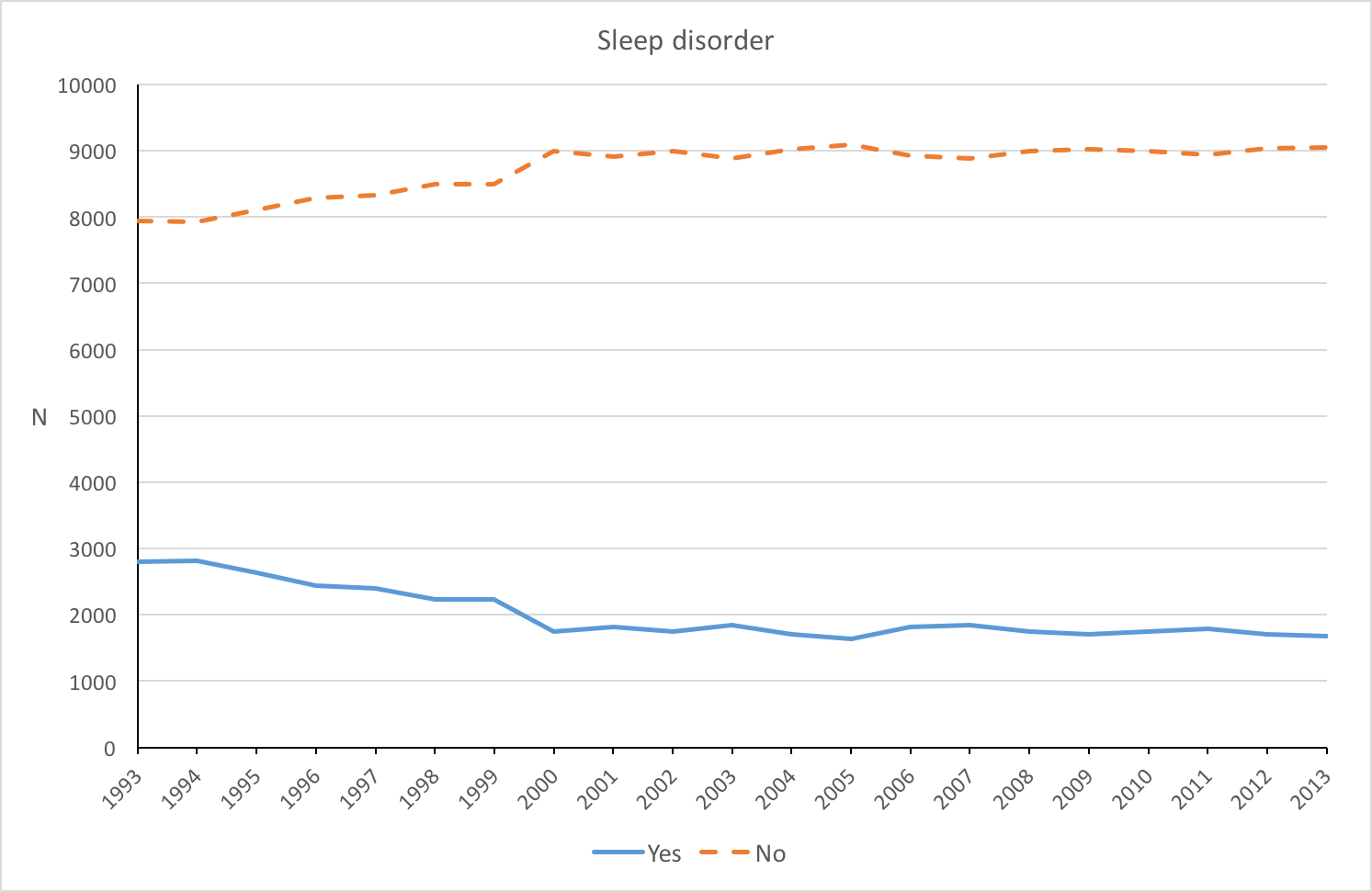


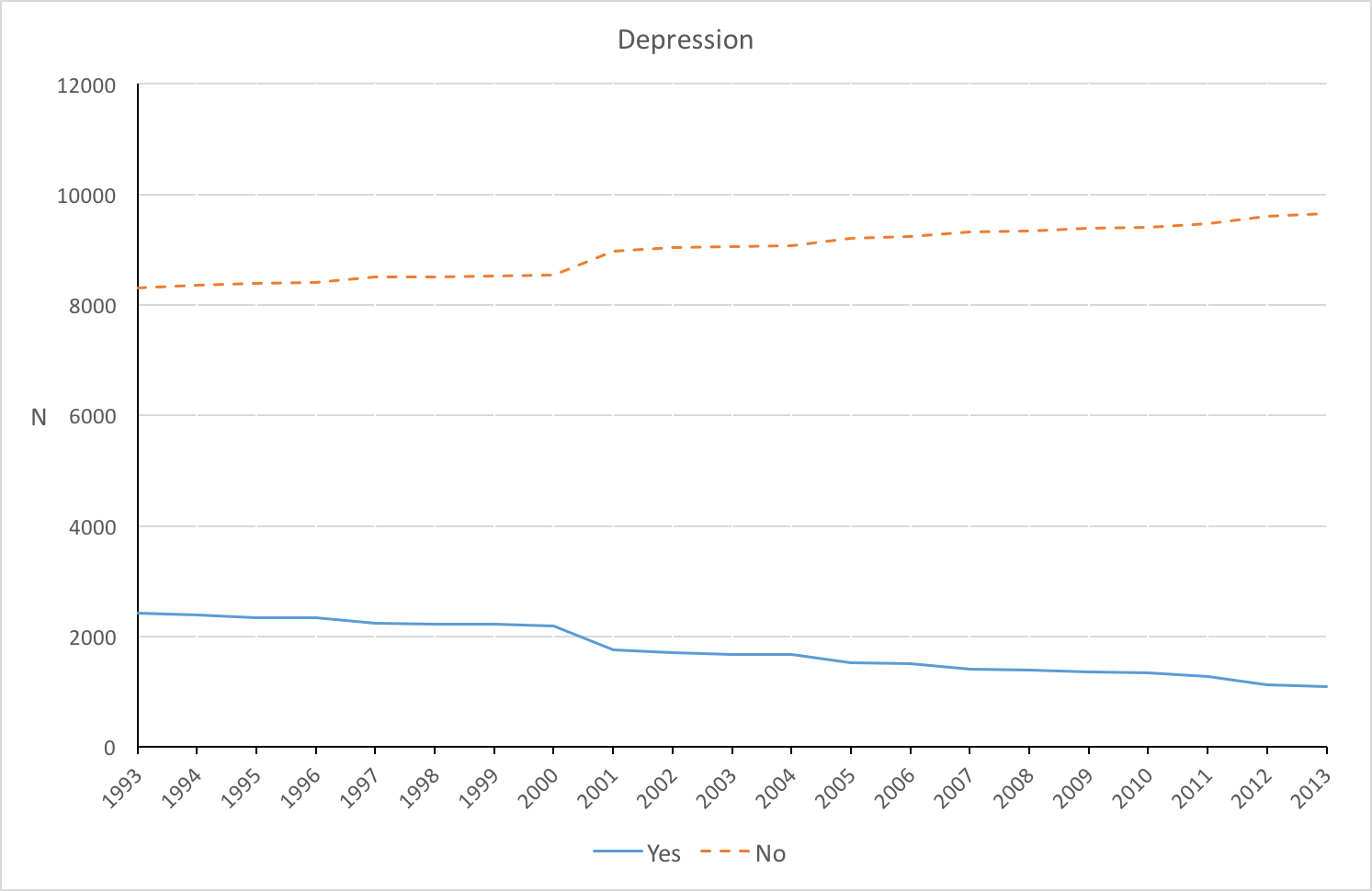


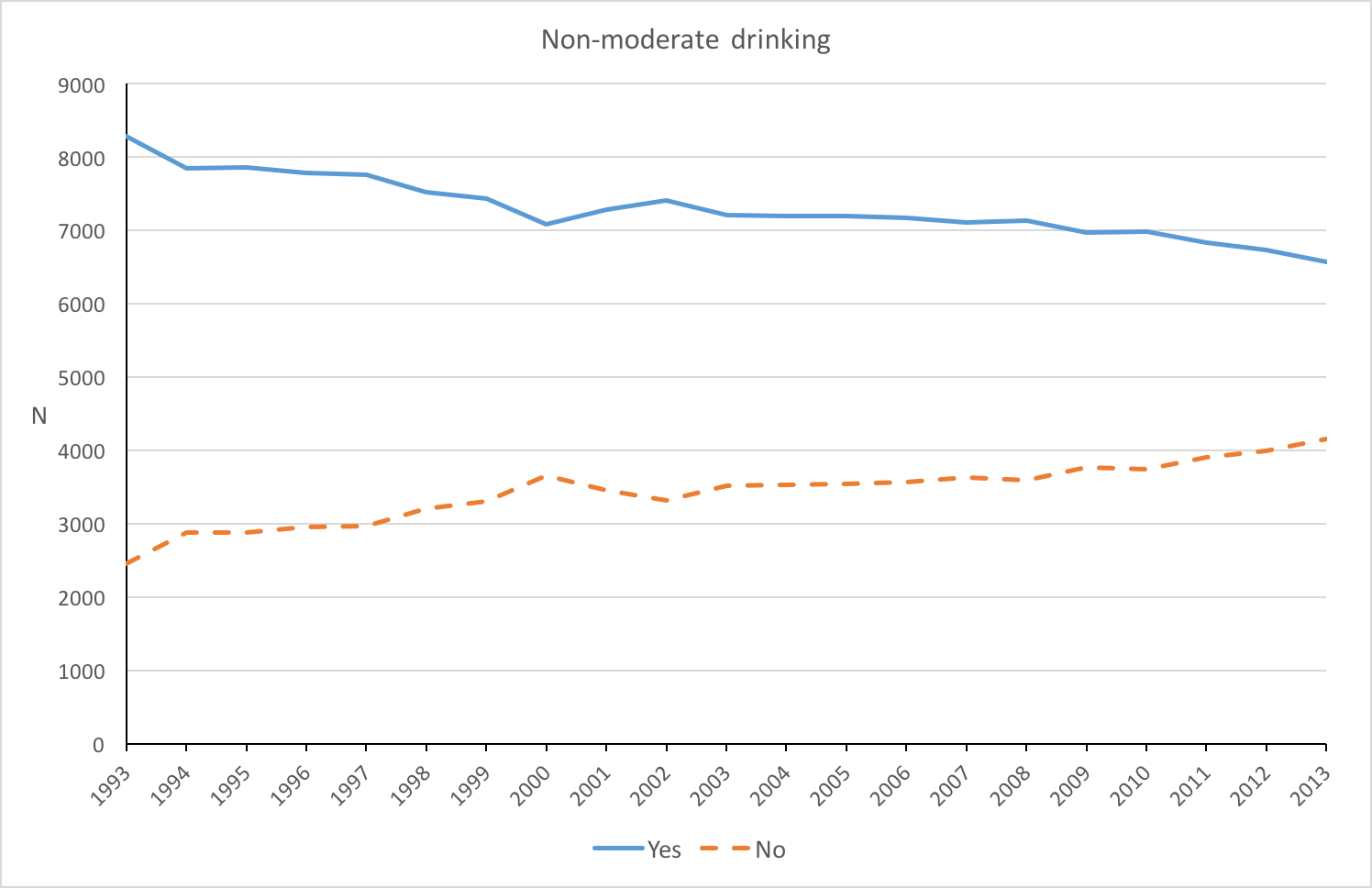


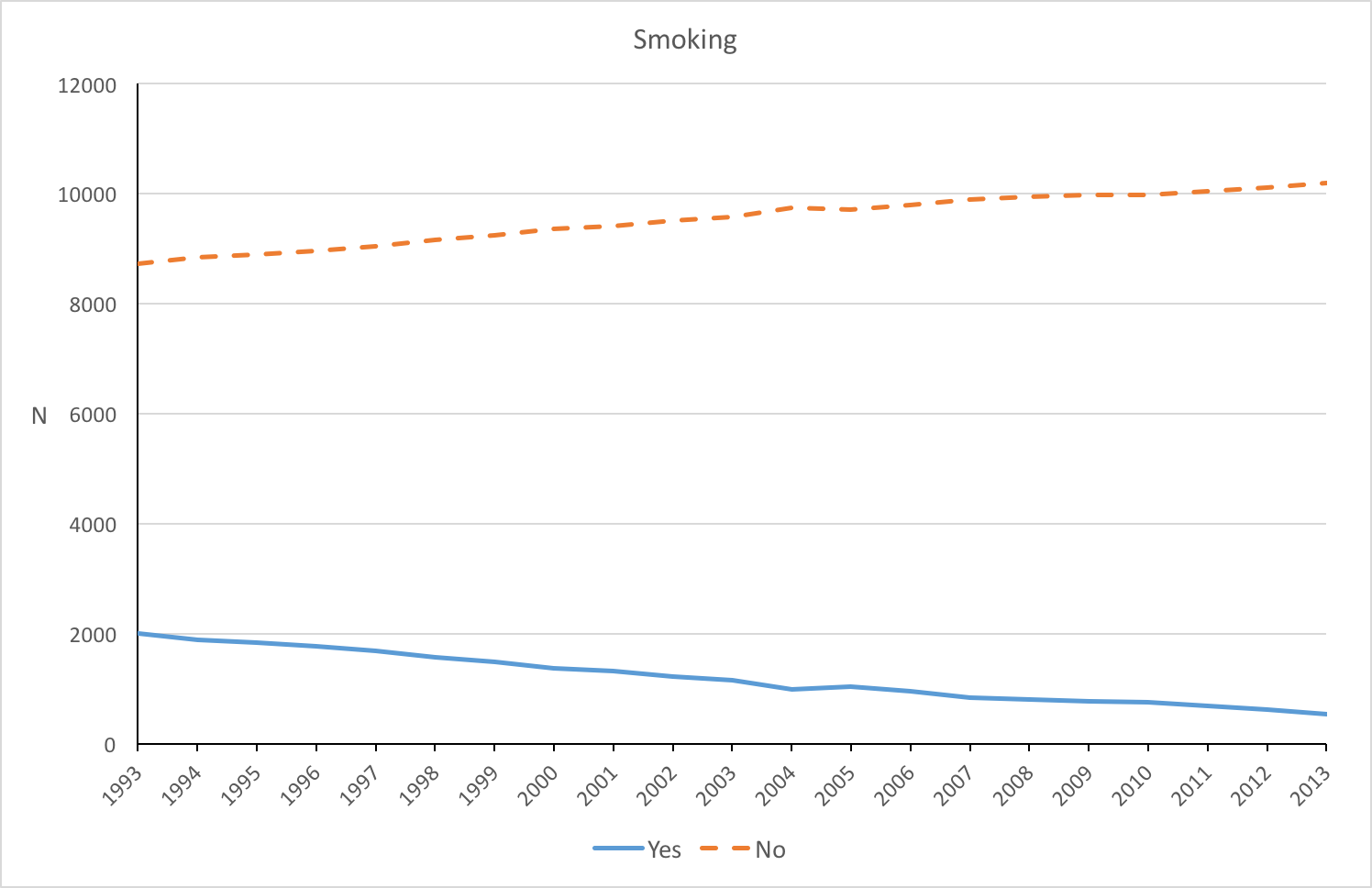


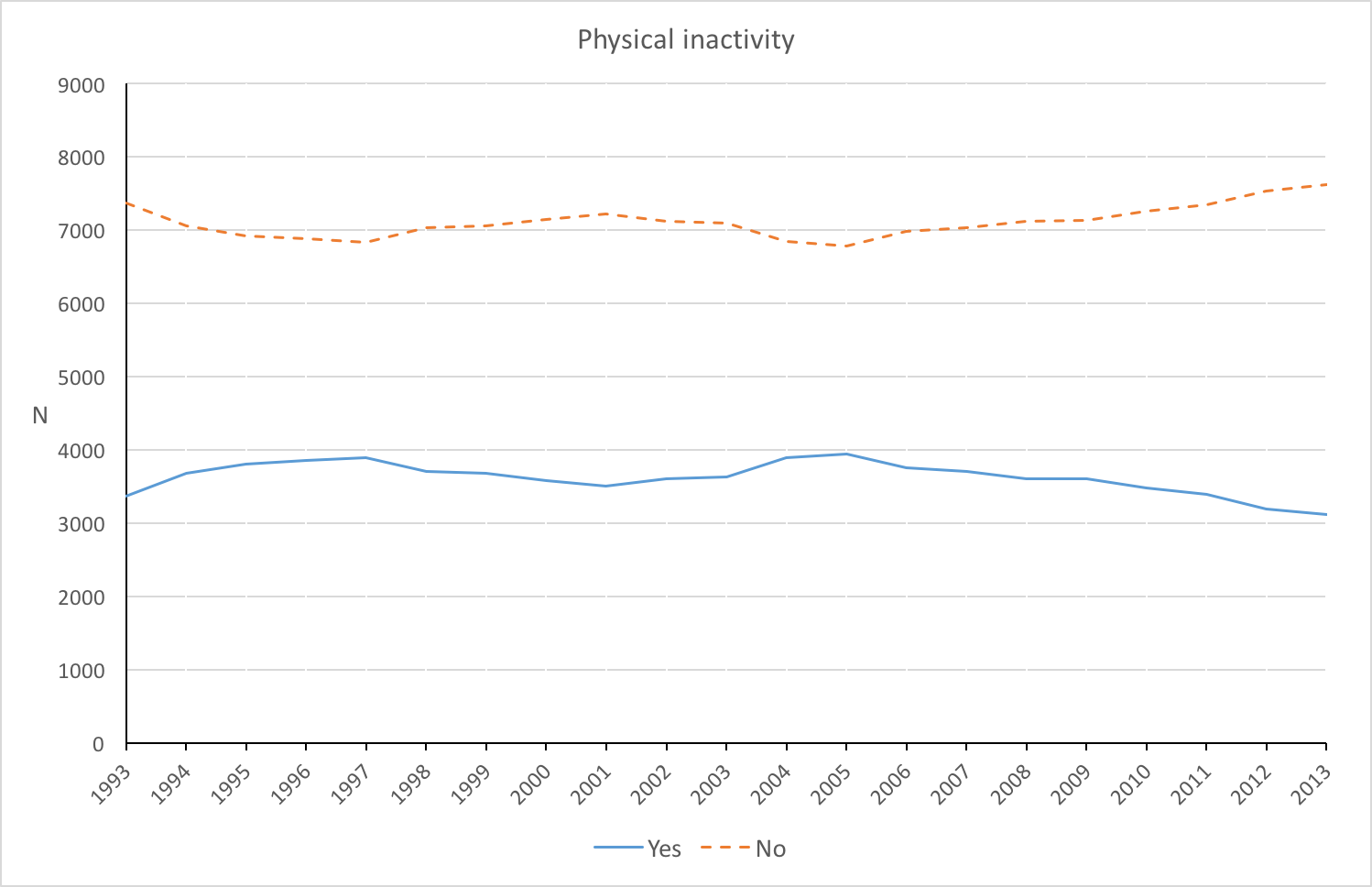

Supplement: S1 Fig — (DOCX) [file pone.0162386.s001.docx]
