# Supplementary material for: A Global View of the Relationships between the Main Behavioural and Clinical Cardiovascular Risk Factors in the GAZEL Prospective Cohort
Source: PLoS One. 2016 Sep 6;11(9):e0162386. doi: 10.1371/journal.pone.0162386 (PMC5012694; doi:10.1371/journal.pone.0162386)
Supplement: S2 Fig — (DOCX) [file pone.0162386.s002.docx]

Diabetes as predictor of CVD events

Gender as predictor of CVD events

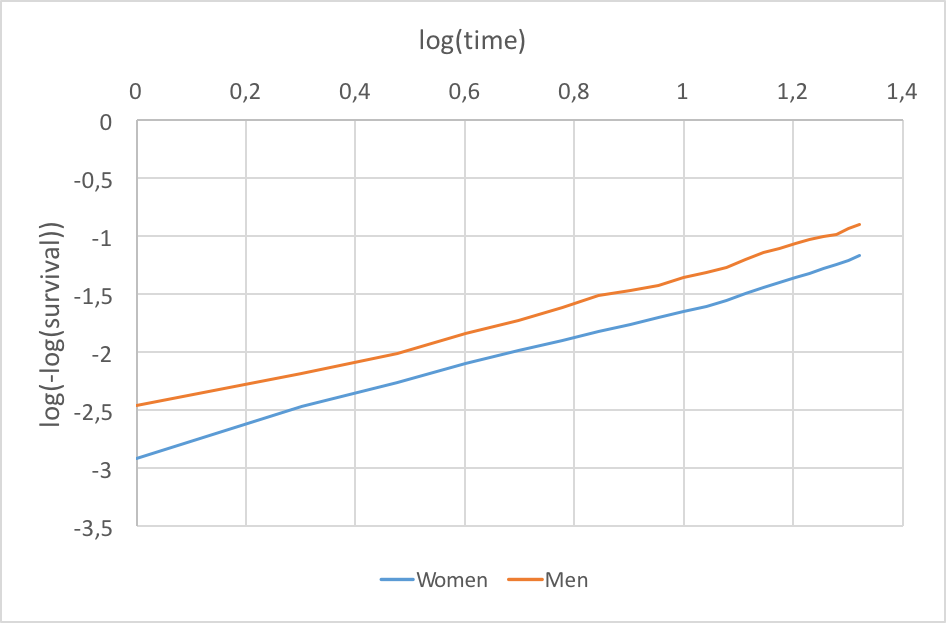


Smoking as predictor of CVD events


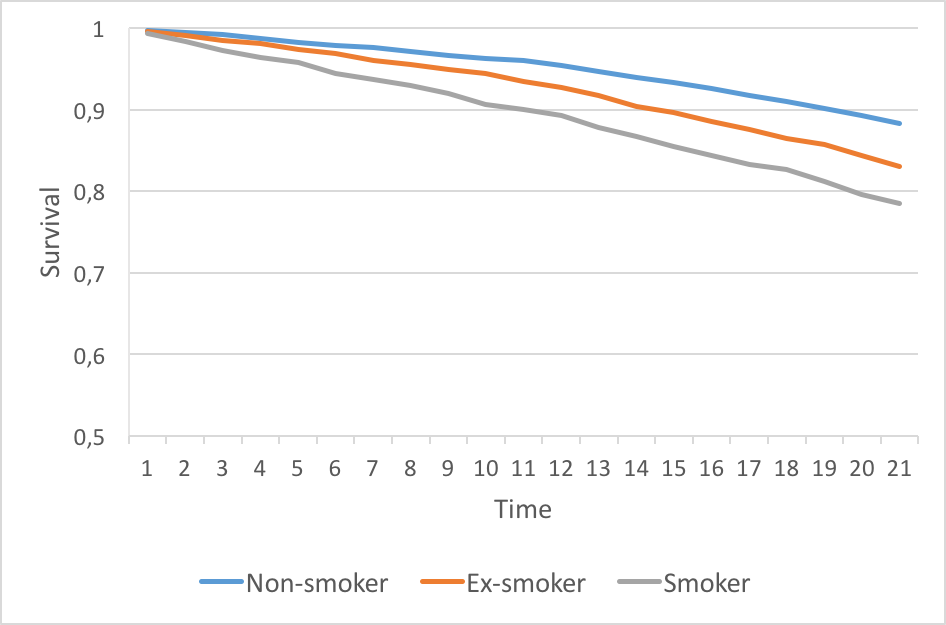


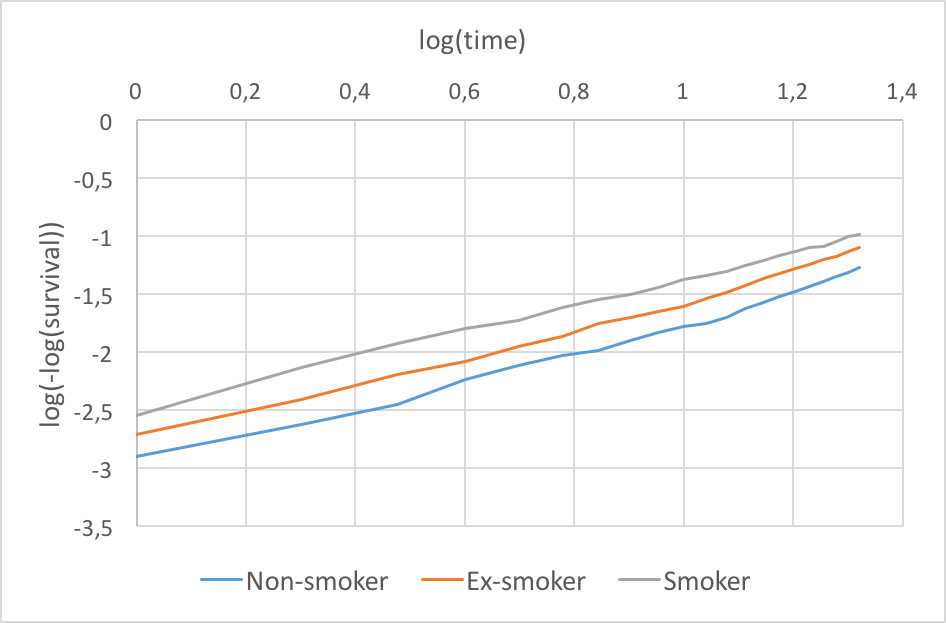


Age as predictor of CVD events

Hypertension as predictor of CVD events

Body mass index as predictor of CVD events


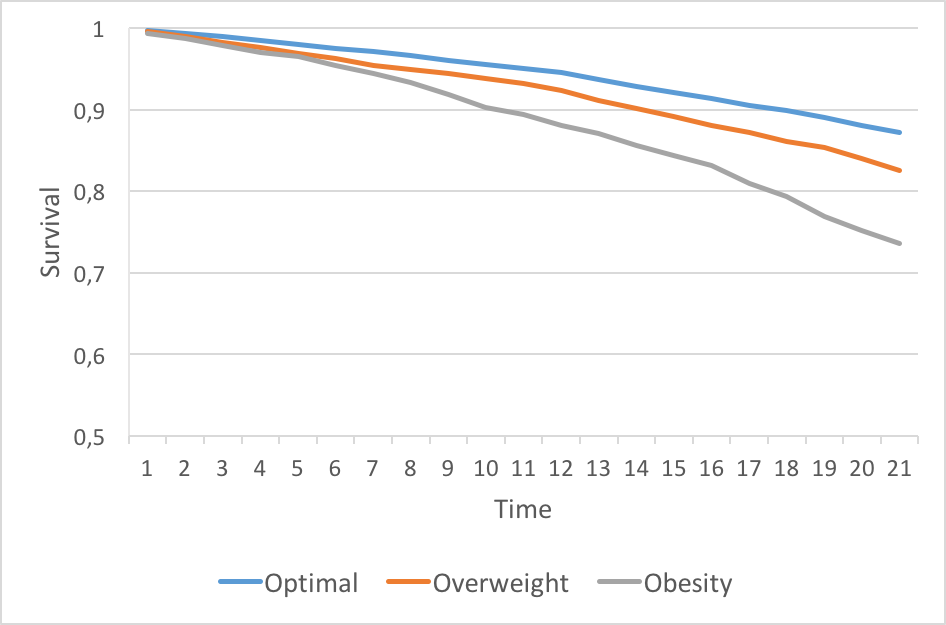


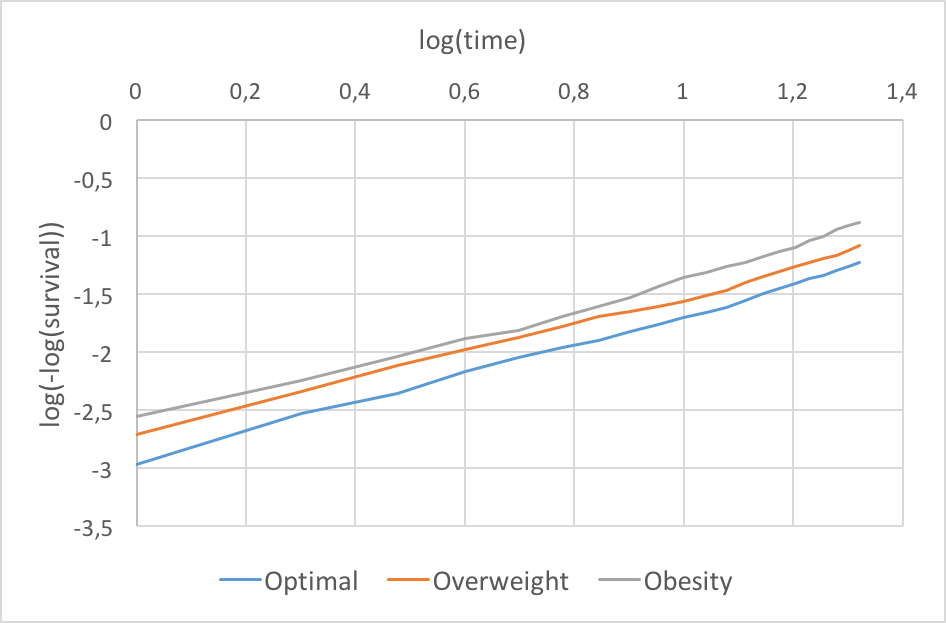


Parental CVD as predictor of CVD events

Alcohol consumption as predictor of CVD events

Sleep disorder as predictor of CVD events

Dyslipidemia as predictor of CVD events

Physical activity as predictor of CVD events


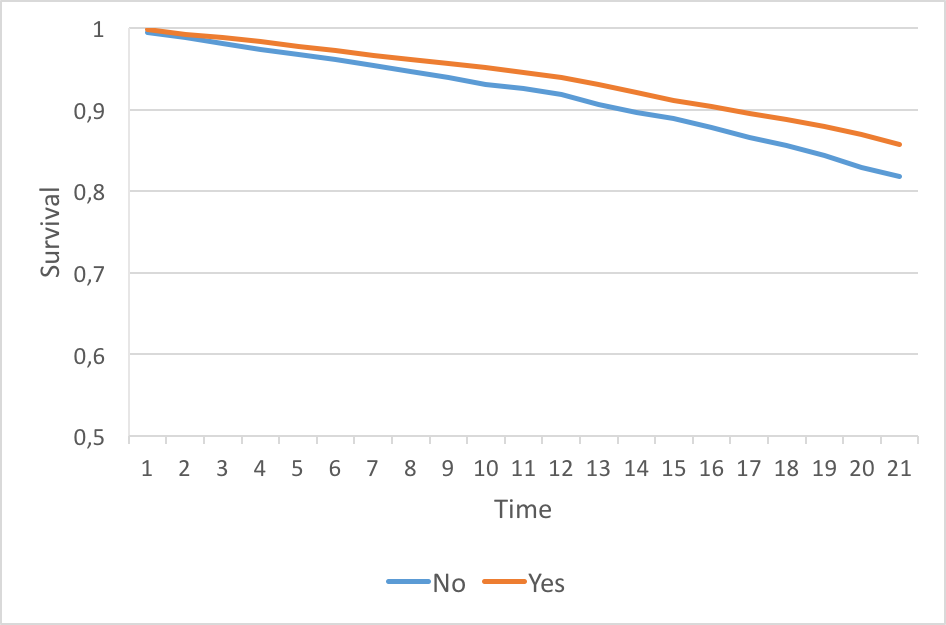


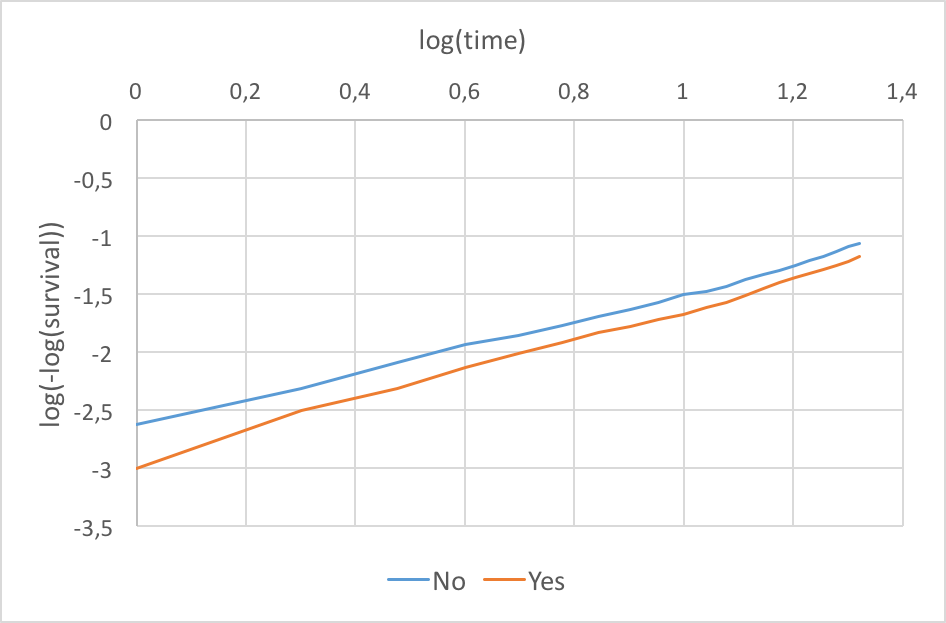


Depression as predictor of CVD events

Smoking as predictor of non-moderate drinking


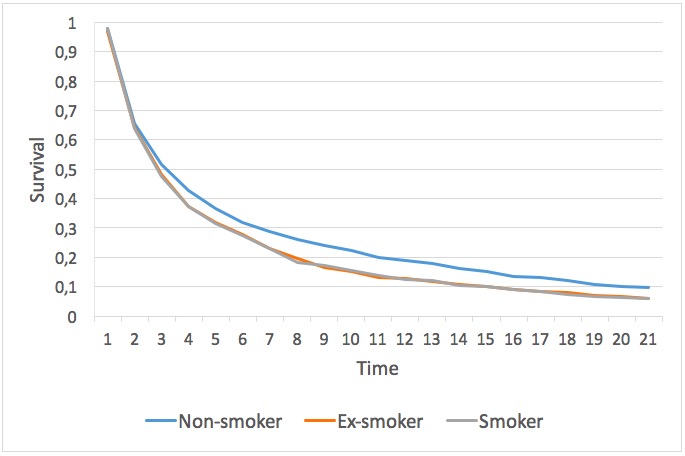

Body mass index as predictor of smoking

Depression as predictor of smoking

Body mass index as predictor of physical inactivity

Smoking as predictor of physical inactivity

Depression as predictor of physical inactivity

Dyslipidemia as predictor of physical inactivity

Gender as predictor of physical inactivity


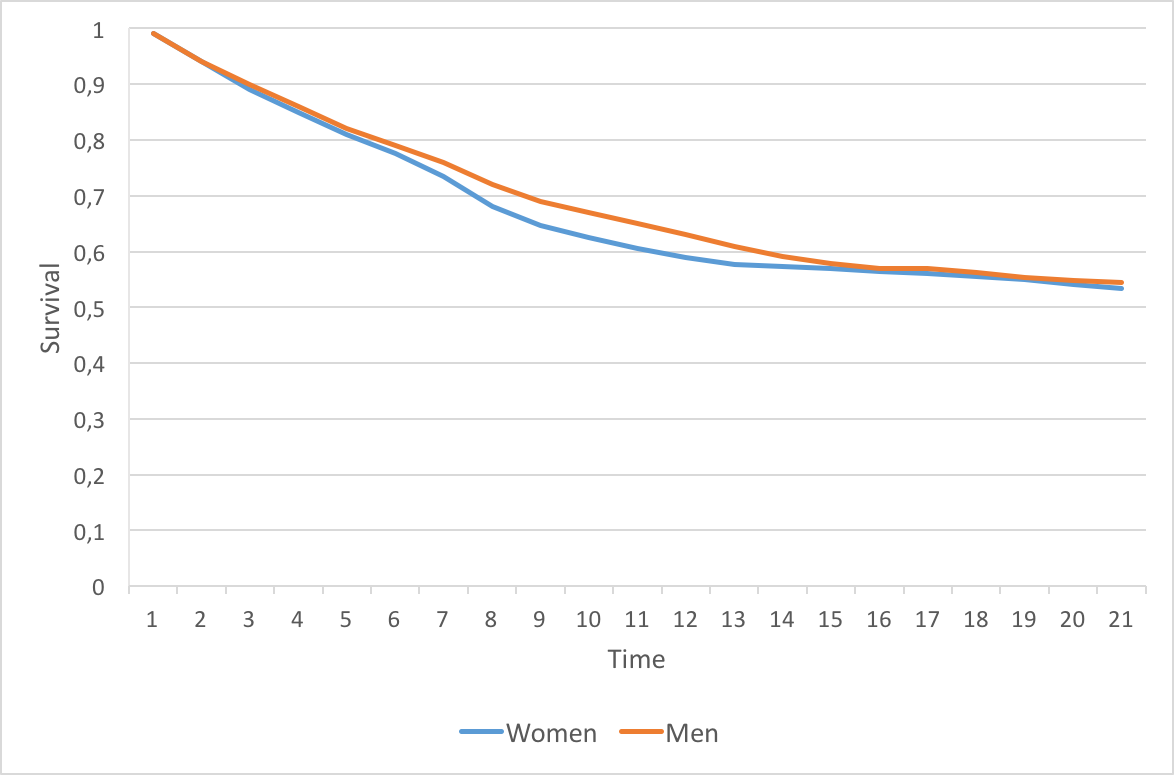


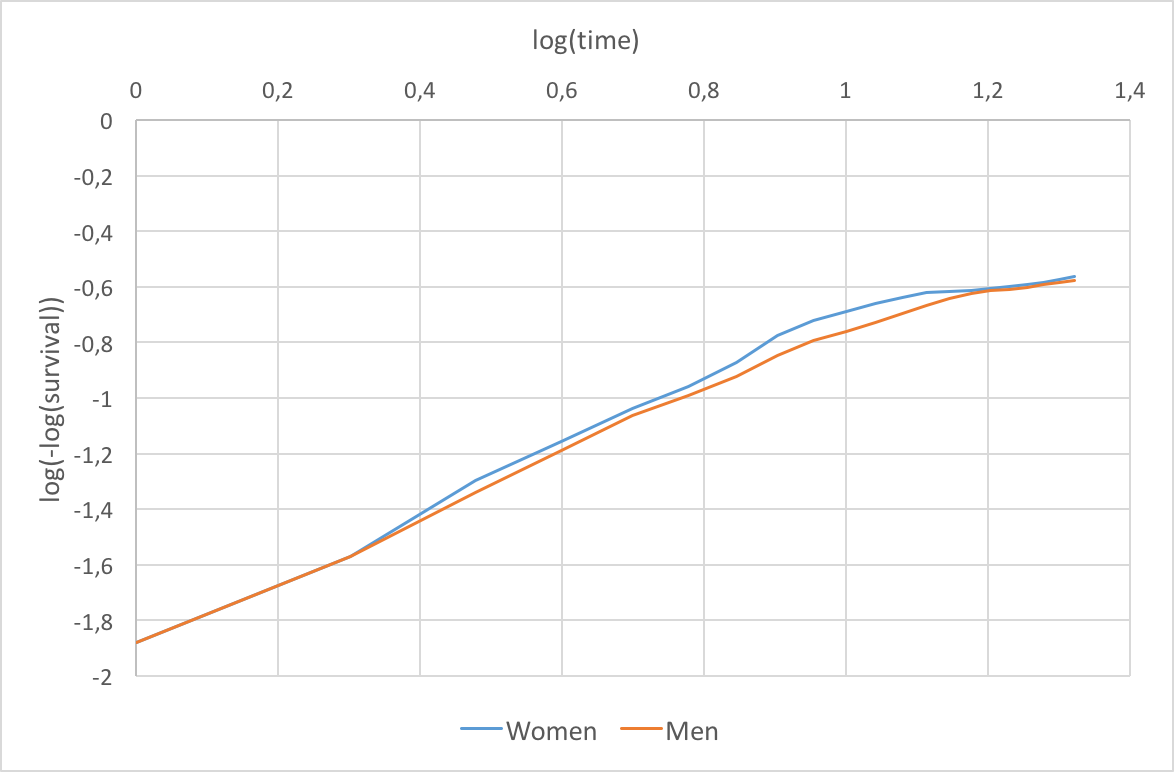


Hypertension as predictor of physical inactivity

Hypertension as predictor of obesity

Smoking as predictor of obesity

Physical activity as predictor of obesity

Dyslipidemia as predictor of obesity

Depression as predictor of obesity

Body mass index as predictor of hypertension

Age as predictor of hypertension

Diabetes as predictor of hypertension

Dyslipidemia as predictor of hypertension

Parental CVD as predictor of hypertension

Sleep disorder as predictor of hypertension

Depression as predictor of hypertension

Physical activity as predictor of hypertension

Hypertension as predictor of dyslipidemia

Body mass index as predictor of dyslipidemia

Diabetes as predictor of dyslipidemia

Age as predictor of dyslipidemia

Smoking as predictor of dyslipidemia

Depression as predictor of dyslipidemia

Sleep disorder as predictor of dyslipidemia

Parental CVD as predictor of dyslipidemia

Physical activity as predictor of dyslipidemia

Body mass index as predictor of diabetes

Hypertension as predictor of diabetes

Dyslipidemia as predictor of diabetes

Smoking as predictor of diabetes

Depression as predictor of diabetes

Parental CVD as predictor of diabetes

Physical activity as predictor of diabetes

Depression as predictor of sleep disorder

Gender as predictor of sleep disorder

Age as predictor of sleep disorder

Parental CVD as predictor of sleep disorder

Smoking as predictor of sleep disorder

Sleep disorder as predictor of depression

Age as predictor of depression

Smoking as predictor of depression

Parental CVD as predictor of depression
