# Supplementary material for: A Global View of the Relationships between the Main Behavioural and Clinical Cardiovascular Risk Factors in the GAZEL Prospective Cohort
Source: PLoS One. 2016 Sep 6;11(9):e0162386. doi: 10.1371/journal.pone.0162386 (PMC5012694; doi:10.1371/journal.pone.0162386)
Supplement: S4 Fig — (DOCX) [file pone.0162386.s004.docx]

Overall pattern of missing data during follow-up for the 12 CVD risk factors included in the analyses.

The figure is a treemap where the number of volunteers with a particular pattern of missing values is represented by the size of the corresponding rectangular area. The 20-digit string in each area represents the 20 years of follow-up read in the same order from left to right; on a particular year, 0 corresponds to the absence of missing values in all the factors, 1 to the presence of missing values in at least one of the factors. The colour of the areas reflects the number of years where missing data are present with a continuous spectrum from bright blue (volunteers without missing values over the entire follow-up) to bright red (volunteers with missing values every year).
