# Supplementary material for: A Global View of the Relationships between the Main Behavioural and Clinical Cardiovascular Risk Factors in the GAZEL Prospective Cohort
Source: PLoS One. 2016 Sep 6;11(9):e0162386. doi: 10.1371/journal.pone.0162386 (PMC5012694; doi:10.1371/journal.pone.0162386)
Supplement: S1 Table — (DOCX) [file pone.0162386.s005.docx]

Risk of CVD events according to predictive factors at baseline - multi-adjusted associations without excluding loss to follow-up volunteers.

|  | | **HR (95% CI)** | **p** |
| --- | --- | --- | --- |
| **Diabetes** | No  Yes | 1.00  1.98 (1.49-2.64) | <0.0001 |
| **Gender** | Women | 1.00 |  |
|  | Men | 1.92 (1.63-2.25) | <0.0001 |
| **Smoking** | Non-smoker | 1.00 |  |
|  | Ex-smoker | 1.25 (1.10-1.41) | 0.0004 |
|  | Smoker | 1.80 (1.57-2.07) | <0.0001 |
| **Age (y)** | 39-45 | 1.00 |  |
|  | 46-49 | 1.26 (1.10-1.44) | 0.001 |
|  | 50-54 | 1.57 (1.38-1.79) | <0.0001 |
| **Hypertension** | No  Yes | 1.00  1.58 (1.37-1.83) | <0.0001 |
| **Body mass index** | Optimal | 1.00 |  |
|  | Overweight | 1.09 (0.98-1.22) | 0.12 |
|  | Obesity | 1.56 (1.29-1.87) | <0.0001 |
| **Parental CVD** | No | 1.00 |  |
|  | Yes | 1.46 (1.27-1.67) | <0.0001 |
| **Alcohol consumption** | Non-drinker | 1.00 |  |
|  | Light drinker | 0.89 (0.75-1.05) | 0.18 |
|  | Moderate drinker | 0.77 (0.64-0.93) | 0.007 |
|  | Heavy drinker | 0.88 (0.72-1.08) | 0.23 |
| **Sleep disorder** | No  Yes | 1.00  1.24 (1.10-1.39) | 0.0003 |
| **Dyslipidemia** | No  Yes | 1.00  1.19 (1.05-1.35) | 0.007 |
| **Physical activity** | No | 1.00 |  |
|  | Yes | 0.82 (0.74-0.92) | 0.0004 |
| **Depression** | No  Yes | 1.00  1.17 (1.04-1.32) | 0.008 |

Risk of incident CVD factors according to the same factors at baseline - summary of multi-adjusted associations without excluding loss to follow-up volunteers.

| **Predictive factors** | | **Incident factors** | | | | | | | | |
| --- | --- | --- | --- | --- | --- | --- | --- | --- | --- | --- |
|  |  | **Diabetes** | **Smoking** | **Hypertension** | **Obesity** | **Non-moderate**  **alcohol**  **consumption** | **Sleep**  **disorder** | **Dyslipidemia** | **Physical**  **inactivity** | **Depression** |
| **Diabetes** | No | - | NS | 1.00 | NS | NS | NS | 1.00  1.36 (1.01-1.89) | NS | NS |
|  | Yes |  |  | 1.41 (1.06-1.87) |  |  |  |  |  |  |
| **Gender** | Women | NS | NS | NS | NS | NS | 1.00 | NS | 1.00 | NS |
|  | Men |  |  |  |  |  | 0.53 (0.48-0.58) |  | 0.92 (0.84-0.99) |  |
| **Smoking** | Non-smoker | 1.00 |  | NS | 1.00 | 1.00 | 1.00  1.01 (0.92-1.10)  1.20 (1.08-1.34) | 1.00 | 1.00 | 1.00 |
|  | Ex-smoker | 1.21 (1.03-1.42) | - |  | 1.50 (1.32-1.69) | 1.16 (1.05-1.27) |  | 1.11 (1.02-1.21) | 0.99 (0.91-1.07) | 1.05 (0.96-1.15) |
|  | Smoker | 1.53 (1.27-1.85) |  |  | 1.61 (1.39-1.86) | 1.22 (1.09-1.36) |  | 1.25 (1.13-1.38) | 1.35 (1.23-1.49) | 1.31 (1.17-1.46) |
| **Age (y)** | 39-45 | NS | NS | 1.00 | NS | NS | 1.00 | 1.00 | NS | 1.00 |
|  | 46-49 |  |  | 1.20 (1.09-1.32) |  |  | 1.03 (0.93-1.13) | 1.18 (1.08-1.29) |  | 0.93 (0.85-1.03) |
|  | 50-54 |  |  | 1.30 (1.19-1.43) |  |  | 0.89 (0.80-0.98) | 1.25 (1.14-1.36) |  | 0.83 (0.75-0.92) |
| **Hypertension** | No  Yes | 1.00  1.85 (1.54-2.21) | NS | - | 1.00  1.88 (1.61-2.20) | NS | NS | 1.00  1.52 (1.35-1.71) | 1.00  1.21 (1.07-1.37) | NS |
| **Body mass index** | Optimal | 1.00 | 1.00 | 1.00 |  | NS | NS | 1.00 | 1.00 | NS |
|  | Overweight | 2.41 (2.03-2.85) | 1.28 (1.07-1.52) | 1.68 (1.55-1.82) | - |  |  | 1.26 (1.17-1.36) | 1.21 (1.12-1.31) |  |
|  | Obesity | 7.02 (5.69-8.67) | 1.43 (1.02-2.00) | 2.73 (2.36-3.16) |  |  |  | 1.35 (1.15-1.58) | 1.68 (1.44-1.96) |  |
| **Parental CVD** | No | 1.00 | NS | 1.00 | NS | NS | 1.00 | 1.00 | NS | 1.00 |
|  | Yes | 1.33 (1.10-1.60) |  | 1.22 (1.10-1.36) |  |  | 1.13 (1.01-1.28) | 1.14 (1.02-1.27) |  | 1.20 (1.07-1.35) |
| **Alcohol consumption** | Non-drinker | 1.00 | NS | NS | NS | - | NS | NS | NS | NS |
|  | Light drinker | 0.72 (0.58-0.90) |  |  |  |  |  |  |  |  |
|  | Moderate drinker | 0.71 (0.56-0.91) |  |  |  |  |  |  |  |  |
|  | Heavy drinker | 0.96 (0.74-1.25) |  |  |  |  |  |  |  |  |
| **Sleep disorder** | No | NS | NS | 1.00 | NS | NS | - | 1.00 | NS | 1.00 |
|  | Yes |  |  | 1.13 (1.04-1.23) |  |  |  | 1.11 (1.02-1.21) |  | 1.76 (1.61-1.93) |
| **Dyslipidemia** | No  Yes | 1.00  1.64 (1.40-1.92) | NS | 1.00  1.17 (1.06-1.29) | 1.00  1.21 (1.05-1.39) | NS | NS | - | 1.00 | NS |
|  |  |  |  |  |  |  |  |  | 1.15 (1.04-1.27) |  |
| **Physical activity** | No | 1.00 | NS | 1.00 | 1.00 | NS | NS | 1.00 | - | NS |
|  | Yes | 0.82 (0.71-0.94) |  | 0.91 (0.84-0.99) | 0.65 (0.59-0.73) |  |  | 0.91 (0.84-0.98) |  |  |
| **Depression** | No | 1.00 | 1.00 | 1.00 | 1.00 | NS | 1.00 | 1.00 | 1.00 | - |
|  | Yes | 1.26 (1.08-1.48) | 1.21 (1.01-1.46) | 1.13 (1.04-1.24) | 1.23 (1.08-1.39) |  | 1.75 (1.58-1.93) | 1.15 (1.06-1.25) | 1.16 (1.06-1.26) |  |

HRs (95% CI). White background: p<0.05, light gray: p<0.01, middle gray: p<0.001, dark gray: p<0.0001, NS: non-significant.
