# Supplementary material for: A Global View of the Relationships between the Main Behavioural and Clinical Cardiovascular Risk Factors in the GAZEL Prospective Cohort
Source: PLoS One. 2016 Sep 6;11(9):e0162386. doi: 10.1371/journal.pone.0162386 (PMC5012694; doi:10.1371/journal.pone.0162386)
Supplement: S2 Table — (DOCX) [file pone.0162386.s006.docx]

Comparison of baseline behavioural and clinical characteristics between the volunteers included in the study and those excluded due to missing values.

|  |  | **Included**  **n (%)** | **Excluded**  **n (%)** | **P** |
| --- | --- | --- | --- | --- |
| **Gender** | Women | 2723 (25.4) | 2876 (29.4) | <0.0001 |
|  | Men | 8013 (74.6) | 6902 (70.6) |  |
| **Age (y)** | 39-45 | 3542 (33.0) | 3353 (34.3) | 0.14 |
|  | 46-49 | 3547 (33.0) | 3174 (32.5) |  |
|  | 50-54 | 3647 (34.0) | 3251 (33.2) |  |
| **Parental CVD** | No | 9437 (87.9) | 4082 (87.4) | 0.34 |
|  | Yes | 1299 (12.1) | 591 (12.6) |  |
| **Alcohol consumption** | Non-drinker | 1277 (11.9) | 709 (13.4) | 0.009 |
|  | Light drinker | 5659 (52.7) | 2662 (50.3) |  |
|  | Moderate drinker | 2454 (22.9) | 1223 (23.1) |  |
|  | Heavy drinker | 1346 (12.5) | 696 (13.2) |  |
| **Smoking** | Non-smoker | 4694 (43.7) | 2092 (41.2) | <0.0001 |
|  | Ex-smoker | 4035 (37.6) | 1804 (35.5) |  |
|  | Smoker | 2007 (18.7) | 1180 (23.3) |  |
| **Physical activity** | No | 3365 (31.3) | 2319 (36.7) | <0.0001 |
|  | Yes | 7371 (68.7) | 4000 (63.3) |  |
| **Body mass index** | Optimal | 5762 (53.7) | 2051 (52.5) | 0.64 |
|  | Overweight | 4328 (40.3) | 1579 (40.5) |  |
|  | Obesity | 646 (6.0) | 275 (7.0) |  |
| **Hypertension** | No  Yes | 9717 (90.5)  1019 (9.5) | 2938 (83.4)  583 (16.6) | <0.0001 |
| **Dyslipidemia** | No  Yes | 9030 (84.1)  1706 (15.9) | 3288 (80.5)  798 (19.5) | <0.0001 |
| **Diabetes** | No  Yes | 10573 (98.5)  163 (1.5) | 941 (89.9)  106 (10.1) | <0.0001 |
| **Sleep disorder** | No  Yes | 7937 (73.9)  2799 (26.1) | 4281 (74.7)  1447 (25.3) | 0.26 |
| **Depression** | No  Yes | 8314 (77.4)  2422 (22.6) | 2951 (71.0)  1206 (29.0) | <0.0001 |

Univariate comparisons were performed with chi-square test (or Fisher’s exact test when necessary).
