# Supplementary material for: A Global View of the Relationships between the Main Behavioural and Clinical Cardiovascular Risk Factors in the GAZEL Prospective Cohort
Source: PLoS One. 2016 Sep 6;11(9):e0162386. doi: 10.1371/journal.pone.0162386 (PMC5012694; doi:10.1371/journal.pone.0162386)
Supplement: S3 Table — (DOCX) [file pone.0162386.s007.docx]

Risk of CVD events according to predictive factors at baseline - multi-adjusted associations with weighting.

|  | | **HR (95% CI)** | **p** |
| --- | --- | --- | --- |
| **Diabetes** | No  Yes | 1.00  2.02 (1.56-2.56) | <0.0001 |
| **Gender** | Women | 1.00 |  |
|  | Men | 1.88 (1.64-2.16) | <0.0001 |
| **Smoking** | Non-smoker | 1.00 |  |
|  | Ex-smoker | 1.23 (1.11-1.37) | 0.0001 |
|  | Smoker | 1.77 (1.57-1.99) | <0.0001 |
| **Age (y)** | 39-45 | 1.00 |  |
|  | 46-49 | 1.23 (1.09-1.39) | 0.0007 |
|  | 50-54 | 1.53 (1.36-1.72) | <0.0001 |
| **Hypertension** | No  Yes | 1.00  1.53 (1.35-1.73) | <0.0001 |
| **Body mass index** | Optimal | 1.00 |  |
|  | Overweight | 1.07 (0.97-1.18) | 0.18 |
|  | Obesity | 1.51 (1.29-1.76) | <0.0001 |
| **Parental CVD** | No | 1.00 |  |
|  | Yes | 1.48 (1.31-1.66) | <0.0001 |
| **Alcohol consumption** | Non-drinker | 1.00 |  |
|  | Light drinker | 0.91 (0.79-1.06) | 0.22 |
|  | Moderate drinker | 0.78 (0.66-0.92) | 0.004 |
|  | Heavy drinker | 0.90 (0.75-1.07) | 0.23 |
| **Sleep disorder** | No  Yes | 1.00  1.27 (1.15-1.40) | <0.0001 |
| **Dyslipidemia** | No  Yes | 1.00  1.20 (1.07-1.33) | 0.001 |
| **Physical activity** | No | 1.00 |  |
|  | Yes | 0.82 (0.75-0.90) | <0.0001 |
| **Depression** | No  Yes | 1.00  1.17 (1.05-1.29) | 0.003 |

Risk of incident CVD factors according to the same factors at baseline - summary of multi-adjusted associations with weighting.

| **Predictive factors** | | **Incident factors** | | | | | | | | |
| --- | --- | --- | --- | --- | --- | --- | --- | --- | --- | --- |
|  |  | **Diabetes** | **Smoking** | **Hypertension** | **Obesity** | **Non-moderate**  **alcohol**  **consumption** | **Sleep**  **disorder** | **Dyslipidemia** | **Physical**  **inactivity** | **Depression** |
| **Diabetes** | No | - | NS | 1.00 | NS | NS | NS | NS | NS | NS |
|  | Yes |  |  | 1.33 (1.04-1.66) |  |  |  |  |  |  |
| **Gender** | Women | NS | NS | NS | NS | 1.00  1.11 (1.02-1.22) | 1.00 | 1.00  0.86 (0.80-0.93) | 1.00 | NS |
|  | Men |  |  |  |  |  | 0.49 (0.45-0.54) |  | 0.87 (0.81-0.95) |  |
| **Smoking** | Non-smoker | 1.00 |  | NS | 1.00 | 1.00 | NS | 1.00 | 1.00 | 1.00 |
|  | Ex-smoker | 1.19 (1.04-1.36) | - |  | 1.45 (1.30-1.60) | 1.13 (1.04-1.23) |  | 1.09 (1.01-1.17) | 1.00 (0.93-1.07) | 1.02 (0.94-1.11) |
|  | Smoker | 1.61 (1.38-1.84) |  |  | 1.57 (1.40-1.77) | 1.14 (1.03-1.25) |  | 1.23 (1.13-1.33) | 1.36 (1.26-1.48) | 1.22 (1.11-1.35) |
| **Age (y)** | 39-45 | NS | NS | 1.00 | 1.00 | NS | 1.00 | 1.00 | 1.00 | 1.00 |
|  | 46-49 |  |  | 1.15 (1.06-1.24) | 0.98 (0.88-1.09) |  | 1.01 (0.93-1.10) | 1.19 (1.10-1.28) | 0.98 (0.91-1.06) | 0.91 (0.83-0.99) |
|  | 50-54 |  |  | 1.23 (1.14-1.33) | 0.79 (0.71-0.88) |  | 0.84 (0.77-0.91) | 1.20 (1.11-1.29) | 0.92 (0.85-0.99) | 0.76 (0.70-0.84) |
| **Hypertension** | No  Yes | 1.00  1.71 (1.49-1.97) | NS | - | 1.00  1.84 (1.62-2.08) | NS | NS | 1.00  1.42 (1.28-1.56) | 1.00  1.14 (1.02-1.26) | NS |
| **Body mass index** | Optimal | 1.00 | 1.00 | 1.00 |  | NS | NS | 1.00 | 1.00 | 1.00 |
|  | Overweight | 2.42 (2.11-2.77) | 1.25 (1.08-1.46) | 1.65 (1.54-1.76) | - |  |  | 1.27 (1.19-1.35) | 1.22 (1.14-1.31) | 1.10 (1.02-1.19) |
|  | Obesity | 6.65 (5.62-7.86) | 1.28 (0.96-1.69) | 2.72 (2.41-3.05) |  |  |  | 1.25 (1.10-1.42) | 1.73 (1.53-1.96) | 1.19 (1.03-1.37) |
| **Parental CVD** | No | 1.00 | NS | 1.00 | 1.00  1.16 (1.02-1.31) | NS | 1.00 | 1.00 | NS | 1.00 |
|  | Yes | 1.29 (1.11-1.49) |  | 1.17 (1.07-1.28) |  |  | 1.12 (1.01-1.24) | 1.11 (1.02-1.22) |  | 1.14 (1.03-1.26) |
| **Alcohol consumption** | Non-drinker | 1.00 | NS | NS | NS | - | NS | NS | NS | NS |
|  | Light drinker | 0.82 (0.69-0.98) |  |  |  |  |  |  |  |  |
|  | Moderate drinker | 0.81 (0.66-0.99) |  |  |  |  |  |  |  |  |
|  | Heavy drinker | 1.03 (0.84-1.27) |  |  |  |  |  |  |  |  |
| **Sleep disorder** | No | NS | NS | 1.00 | NS | NS | - | 1.00 | NS | 1.00 |
|  | Yes |  |  | 1.13 (1.05-1.21) |  |  |  | 1.09 (1.02-1.17) |  | 1.77 (1.63-1.91) |
| **Dyslipidemia** | No  Yes | 1.00  1.66 (1.46-1.88) | NS | 1.00  1.23 (1.14-1.33) | 1.00  1.17 (1.04-1.31) | NS | NS | - | 1.00 | 1.00  1.11 (1.01-1.22) |
|  |  |  |  |  |  |  |  |  | 1.16 (1.07-1.26) |  |
| **Physical activity** | No | 1.00 | NS | 1.00 | 1.00 | NS | NS | 1.00 | - | 1.00  0.92 (0.85-0.99) |
|  | Yes | 0.83 (0.74-0.93) |  | 0.92 (0.87-0.99) | 0.70 (0.64-0.76) |  |  | 0.93 (0.87-0.99) |  |  |
| **Depression** | No | 1.00 | NS | 1.00 | 1.00 | NS | 1.00 | 1.00 | 1.00 | - |
|  | Yes | 1.26 (1.11-1.43) |  | 1.12 (1.05-1.21) | 1.21 (1.10-1.34) |  | 1.77 (1.63-1.92) | 1.12 (1.05-1.21) | 1.17 (1.09-1.25) |  |

HRs (95% CI). White background: p<0.05, light gray: p<0.01, middle gray: p<0.001, dark gray: p<0.0001, NS: non-significant.
