# Supplementary material for: A Global View of the Relationships between the Main Behavioural and Clinical Cardiovascular Risk Factors in the GAZEL Prospective Cohort
Source: PLoS One. 2016 Sep 6;11(9):e0162386. doi: 10.1371/journal.pone.0162386 (PMC5012694; doi:10.1371/journal.pone.0162386)
Supplement: S4 Table — (DOCX) [file pone.0162386.s008.docx]

Risk of CVD events according to predictive factors at baseline - gender-age adjusted associations with complete data for all the risk factors or only for the factor analysed in each model.

|  | | **Complete data for each factor** | | | **Complete data for all the factors** | | |
| --- | --- | --- | --- | --- | --- | --- | --- |
|  |  | **No. of**  **cases** | **HR (95% CI)** | **p** | **No. of**  **cases** | **HR (95% CI)** | **p** |
| **Diabetes** | No  Yes | 2467  69 | 1.00  2.27 (1.77-2.85) | <0.0001 | 1638  56 | 1.00  2.36 (1.79-3.05) | <0.0001 |
| **Gender** | Women | 385 | 1.00 |  | 236 | 1.00 |  |
|  | Men | 2151 | 1.95 (1.74-2.18) | <0.0001 | 1458 | 1.98 (1.72-2.28) | <0.0001 |
| **Smoking** | Non-smoker | 712 | 1.00 |  | 553 | 1.00 |  |
|  | Ex-smoker | 838 | 1.24 (1.12-1.37) | <0.0001 | 710 | 1.34 (1.20-1.50) | <0.0001 |
|  | Smoker | 554 | 1.77 (1.58-1.98) | <0.0001 | 431 | 1.84 (1.62-2.09) | <0.0001 |
| **Age (y)** | 39-45 | 835 | 1.00 |  | 559 | 1.00 |  |
|  | 46-49 | 839 | 1.35 (1.22-1.51) | <0.0001 | 559 | 1.30 (1.14-1.48) | <0.0001 |
|  | 50-54 | 862 | 1.67 (1.51-1.86) | <0.0001 | 576 | 1.67 (1.47-1.89) | <0.0001 |
| **Hypertension** | No  Yes | 2214  322 | 1.00  1.63 (1.45-1.83) | <0.0001 | 1437  257 | 1.00  1.77 (1.55-2.02) | <0.0001 |
| **Body mass index** | Optimal | 863 | 1.00 |  | 748 | 1.00 |  |
|  | Overweight | 900 | 1.21 (1.09-1.33) | 0.0001 | 779 | 1.20 (1.08-1.33) | 0.0007 |
|  | Obesity | 195 | 1.89 (1.61-2.21) | <0.0001 | 167 | 1.90 (1.60-2.25) | <0.0001 |
| **Parental CVD** | No | 1838 | 1.00 |  | 1417 | 1.00 |  |
|  | Yes | 369 | 1.56 (1.39-1.74) | <0.0001 | 277 | 1.52 (1.34-1.73) | <0.0001 |
| **Alcohol consumption** | Non-drinker | 245 | 1.00 |  | 197 | 1.00 |  |
|  | Light drinker | 1092 | 0.86 (0.74-0.99) | 0.03 | 879 | 0.83 (0.71-0.97) | 0.02 |
|  | Moderate drinker | 468 | 0.81 (0.70-0.95) | 0.01 | 358 | 0.75 (0.63-0.90) | 0.002 |
|  | Heavy drinker | 326 | 1.01 (0.85-1.20) | 0.90 | 260 | 0.97 (0.80-1.17) | 0.73 |
| **Sleep disorder** | No  Yes | 1894  642 | 1.00  1.29 (1.18-1.41) | <0.0001 | 1189  505 | 1.00  1.34 (1.21-1.49) | <0.0001 |
| **Dyslipidemia** | No  Yes | 2084  452 | 1.00  1.34 (1.21-1.48) | <0.0001 | 1320  374 | 1.00  1.42 (1.26-1.59) | <0.0001 |
| **Physical activity** | No | 839 | 1.00 |  | 623 | 1.00 |  |
|  | Yes | 1375 | 0.71 (0.66-0.78) | <0.0001 | 1071 | 0.72 (0.66-0.80) | <0.0001 |
| **Depression** | No  Yes | 1458  594 | 1.00  1.39 (1.27-1.53) | <0.0001 | 1237  457 | 1.00  1.34 (1.20-1.49) | <0.0001 |

Risk of incident CVD factors according to the same factors at baseline - summary of gender-age adjusted associations in volunteers with complete data for the risk factor analysed in each model.

| **Predictive factors** | | **Incident factors** | | | | | | | | |
| --- | --- | --- | --- | --- | --- | --- | --- | --- | --- | --- |
|  |  | **Diabetes** | **Smoking** | **Hypertension** | **Obesity** | **Non-moderate**  **alcohol**  **consumption** | **Sleep**  **disorder** | **Dyslipidemia** | **Physical**  **inactivity** | **Depression** |
| **Diabetes** | No | - | NS | 1.00 | NS | 1.00  1.41 (1.06-1.82) | NS | NS | NS | NS |
|  | Yes |  |  | 1.60 (1.27-1.98) |  |  |  |  |  |  |
| **Gender** | Women | 1.00  1.57 (1.39-1.77) | NS | 1.00  1.16 (1.09-1.24) | 1.00  1.16 (1.06-1.27) | 1.00  1.26 (1.17-1.36) | 1.00 | NS | NS | 1.00  0.92 (0.85-0.99) |
|  | Men |  |  |  |  |  | 0.53 (0.50-0.56) |  |  |  |
| **Smoking** | Non-smoker | 1.00 |  | 1.00 | 1.00 | 1.00 | 1.00 | 1.00 | 1.00 | 1.00 |
|  | Ex-smoker | 1.50 (1.33-1.71) | - | 1.19 (1.11-1.27) | 1.53 (1.37-1.71) | 1.10 (1.02-1.19) | 0.99 (0.92-1.07) | 1.15 (1.07-1.22) | 1.06 (0.99-1.14) | 1.08 (1.00-1.17) |
|  | Smoker | 1.74 (1.51-2.01) |  | 1.11 (1.02-1.20) | 1.59 (1.40-1.81) | 1.06 (0.97-1.16) | 1.09 (1.01-1.19) | 1.27 (1.17-1.37) | 1.38 (1.28-1.50) | 1.21 (1.10-1.32) |
| **Age (y)** | 39-45 | 1.00 | NS | 1.00 | 1.00 | 1.00 | 1.00 | 1.00 | NS | 1.00 |
|  | 46-49 | 1.20 (1.06-1.35) |  | 1.18 (1.10-1.27) | 1.02 (0.92-1.12) | 1.06 (0.98-1.15) | 0.97 (0.90-1.04) | 1.16 (1.09-1.24) |  | 0.93 (0.86-1.01) |
|  | 50-54 | 1.26 (1.12-1.42) |  | 1.32 (1.24-1.42) | 0.88 (0.80-0.97) | 1.17 (1.09-1.27) | 0.86 (0.80-0.93) | 1.22 (1.14-1.30) |  | 0.83 (0.76-0.90) |
| **Hypertension** | No  Yes | 1.00  2.32 (2.04-2.63) | NS | - | 1.00  1.92 (1.70-2.15) | 1.00  1.47 (1.31-1.64) | NS | 1.00  1.46 (1.32-1.60) | 1.00  1.34 (1.22-1.48) | 1.00  1.22 (1.09-1.37) |
| **Body mass index** | Optimal | 1.00 | 1.00 | 1.00 |  | NS | NS | 1.00 | 1.00 | 1.00 |
|  | Overweight | 2.67 (2.33-3.06) | 1.28 (1.10-1.48) | 1.66 (1.55-1.78) | - |  |  | 1.28 (1.20-1.36) | 1.24 (1.16-1.32) | 1.08 (1.00-1.17) |
|  | Obesity | 8.44 (7.16-9.93) | 1.41 (1.07-1.83) | 2.91 (2.58-3.26) |  |  |  | 1.29 (1.12-1.46) | 1.70 (1.49-1.94) | 1.20 (1.03-1.39) |
| **Parental CVD** | No | 1.00 | NS | 1.00 | 1.00  1.23 (1.09-1.38) | NS | 1.00 | 1.00 | NS | 1.00 |
|  | Yes | 1.44 (1.25-1.65) |  | 1.23 (1.13-1.33) |  |  | 1.12 (1.02-1.22) | 1.18 (1.09-1.28) |  | 1.12 (1.01-1.24) |
| **Alcohol consumption** | Non-drinker | 1.00 | NS | 1.00 | 1.00 | - | NS | 1.00 | 1.00 | 1.00 |
|  | Light drinker | 0.75 (0.63-0.88) |  | 0.94 (0.86-1.04) | 0.98 (0.86-1.13) |  |  | 0.99 (0.90-1.09) | 0.95 (0.86-1.05) | 0.90 (0.81-1.00) |
|  | Moderate drinker | 0.80 (0.67-0.96) |  | 0.98 (0.88-1.09) | 0.90 (0.78-1.05) |  |  | 1.04 (0.94-1.15) | 1.01 (0.91-1.13) | 0.88 (0.78-0.99) |
|  | Heavy drinker | 1.16 (0.96-1.42) |  | 1.18 (1.04-1.32) | 1.22 (1.03-1.44) |  |  | 1.17 (1.04-1.32) | 1.17 (1.03-1.32) | 1.07 (0.93-1.23) |
| **Sleep disorder** | No | NS | NS | 1.00 | NS | 1.00  1.54 (1.43-1.65) | - | 1.00 | 1.00  1.18 (1.11-1.26) | 1.00 |
|  | Yes |  |  | 1.16 (1.09-1.24) |  |  |  | 1.12 (1.05-1.19) |  | 1.85 (1.71-1.99) |
| **Dyslipidemia** | No  Yes | 1.00  1.88 (1.68-2.10) | NS | 1.00  1.37 (1.27-1.48) | 1.00  1.36 (1.22-1.50) | 1.00  1.59 (1.45-1.74) | 1.00  1.14 (1.04-1.24) | - | 1.00 | 1.00  1.27 (1.16-1.39) |
|  |  |  |  |  |  |  |  |  | 1.25 (1.16-1.36) |  |
| **Physical activity** | No | 1.00 | NS | 1.00 | 1.00 | NS | NS | 1.00 | - | 1.00  0.91 (0.85-0.98) |
|  | Yes | 0.65 (0.58-0.72) |  | 0.86 (0.81-0.92) | 0.66 (0.61-0.72) |  |  | 0.90 (0.85-0.95) |  |  |
| **Depression** | No | 1.00 | 1.00 | 1.00 | 1.00 | NS | 1.00 | 1.00 | 1.00 | - |
|  | Yes | 1.35 (1.21-1.52) | 1.21 (1.04-1.40) | 1.17 (1.09-1.25) | 1.22 (1.11-1.35) |  | 1.76 (1.63-1.91) | 1.15 (1.08-1.23) | 1.21 (1.13-1.30) |  |

HRs (95% CI). White background: p<0.05, light gray: p<0.01, middle gray: p<0.001, dark gray: p<0.0001, NS: non-significant.

Risk of incident CVD factors according to the same factors at baseline - summary of gender-age adjusted associations in volunteers with complete data for all the risk factors.

| **Predictive factors** | | **Incident factors** | | | | | | | | |
| --- | --- | --- | --- | --- | --- | --- | --- | --- | --- | --- |
|  |  | **Diabetes** | **Smoking** | **Hypertension** | **Obesity** | **Non-moderate**  **alcohol**  **consumption** | **Sleep**  **disorder** | **Dyslipidemia** | **Physical**  **inactivity** | **Depression** |
| **Diabetes** | No | - | NS | 1.00 | NS | NS | NS | 1.00  1.53 (1.19-1.94) | NS | NS |
|  | Yes |  |  | 1.53 (1.20-1.93) |  |  |  |  |  |  |
| **Gender** | Women | 1.00  1.59 (1.35-1.89) | NS | 1.00  1.16 (1.06-1.26) | NS | 1.00  1.17 (1.06-1.29) | 1.00 | NS | NS | 1.00  0.90 (0.82-0.98) |
|  | Men |  |  |  |  |  | 0.58 (0.55-0.62) |  |  |  |
| **Smoking** | Non-smoker | 1.00 |  | 1.00 | 1.00 | 1.00 | 1.00  1.06 (0.99-1.13)  1.15 (1.07-1.24) | 1.00 | 1.00 | 1.00 |
|  | Ex-smoker | 1.44 (1.25-1.65) | - | 1.18 (1.09-1.27) | 1.55 (1.39-1.73) | 1.12 (1.02-1.23) |  | 1.13 (1.06-1.21) | 1.05 (0.97-1.13) | 1.06 (0.96-1.16) |
|  | Smoker | 1.74 (1.49-2.04) |  | 1.08 (0.98-1.19) | 1.60 (1.41-1.82) | 1.14 (1.02-1.27) |  | 1.21 (1.12-1.31) | 1.41 (1.29-1.55) | 1.25 (1.13-1.39) |
| **Age (y)** | 39-45 | 1.00 | NS | 1.00 | 1.00 | NS | 1.00 | 1.00 | NS | 1.00 |
|  | 46-49 | 1.14 (0.97-1.34) |  | 1.20 (1.11-1.31) | 1.02 (0.91-1.16) |  | 1.03 (0.96-1.10) | 1.17 (1.09-1.27) |  | 0.93 (0.85-1.03) |
|  | 50-54 | 1.23 (1.05-1.44) |  | 1.37 (1.26-1.48) | 0.86 (0.76-0.98) |  | 0.89 (0.83-0.95) | 1.28 (1.19-1.38) |  | 0.79 (0.71-0.87) |
| **Hypertension** | No  Yes | 1.00  2.62 (2.26-3.02) | NS | - | 1.00  2.03 (1.78-2.31) | NS | NS | 1.00  1.63 (1.48-1.78)) | 1.00  1.24 (1.10-1.40) | NS |
| **Body mass index** | Optimal | 1.00 | 1.00 | 1.00 |  | NS | NS | 1.00 | 1.00 | 1.00  1.13 (1.04-1.23)  1.24 (1.05-1.46) |
|  | Overweight | 2.46 (2.13-2.84) | 1.29 (1.13-1.48) | 1.73 (1.62-1.86) | - |  |  | 1.32 (1.24-1.41) | 1.24 (1.15-1.34) |  |
|  | Obesity | 7.69 (6.46-9.14) | 1.26 (0.97-1.62) | 2.94 (2.61-3.31) |  |  |  | 1.57 (1.38-1.77) | 1.80 (1.56-2.08) |  |
| **Parental CVD** | No | 1.00 | NS | 1.00 | 1.00  1.21 (1.05-1.39) | NS | 1.00 | 1.00 | NS | 1.00 |
|  | Yes | 1.35 (1.14-1.58) |  | 1.26 (1.15-1.38) |  |  | 1.15 (1.06-1.25) | 1.15 (1.05-1.25) |  | 1.17 (1.05-1.31) |
| **Alcohol consumption** | Non-drinker | 1.00 | NS | NS | NS | - | NS | 1.00 | NS | NS |
|  | Light drinker | 0.77 (0.63-0.95) |  |  |  |  |  | 0.94 (0.85-1.05) |  |  |
|  | Moderate drinker | 0.76 (0.61-0.96) |  |  |  |  |  | 0.98 (0.87-1.11) |  |  |
|  | Heavy drinker | 1.14 (0.90-1.45) |  |  |  |  |  | 1.16 (1.01-1.33) |  |  |
| **Sleep disorder** | No | NS | NS | 1.00 | NS | NS | - | 1.00 | 1.00  1.13 (1.04-1.22) | 1.00 |
|  | Yes |  |  | 1.20 (1.12-1.29) |  |  |  | 1.16 (1.08-1.24) |  | 1.78 (1.63-1.94) |
| **Dyslipidemia** | No  Yes | 1.00  2.03 (1.78-2.32) | NS | 1.00  1.36 (1.25-1.48) | 1.00  1.37 (1.21-1.53) | NS | 1.00  1.12 (1.01-1.24) | - | 1.00 | 1.00  1.16 (1.04-1.29) |
|  |  |  |  |  |  |  |  |  | 1.24 (1.13-1.36) |  |
| **Physical activity** | No | 1.00 | NS | 1.00 | 1.00 | NS | NS | 1.00 | - | 1.00  0.89 (0.82-0.97) |
|  | Yes | 0.66 (0.58-0.74) |  | 0.83 (0.78-0.89) | 0.63 (0.57-0.69) |  |  | 0.86 (0.80-0.91) |  |  |
| **Depression** | No | 1.00 | 1.00  1.19 (1.03-1.37) | 1.00 | 1.00 | NS | 1.00 | 1.00 | 1.00 | - |
|  | Yes | 1.37 (1.20-1.56) |  | 1.19 (1.11-1.28) | 1.29 (1.16-1.43) |  | 1.88 (1.77-2.00) | 1.19 (1.11-1.27) | 1.21 (1.11-1.31) |  |

HRs (95% CI). White background: p<0.05, light gray: p<0.01, middle gray: p<0.001, dark gray: p<0.0001, NS: non-significant.
