# Supplementary material for: A Global View of the Relationships between the Main Behavioural and Clinical Cardiovascular Risk Factors in the GAZEL Prospective Cohort
Source: PLoS One. 2016 Sep 6;11(9):e0162386. doi: 10.1371/journal.pone.0162386 (PMC5012694; doi:10.1371/journal.pone.0162386)
Supplement: S5 Table — (DOCX) [file pone.0162386.s009.docx]

Risk of CVD events according to time-independent or time-dependent predictive factors - multi-adjusted associations.

|  | | **Time-independent** | | **Time-dependent** | |
| --- | --- | --- | --- | --- | --- |
|  |  | **HR (95% CI)** | **p** | **HR (95% CI)** | **p** |
| **Diabetes** | No  Yes | 1.00  2.02 (1.50-2.66) | <0.0001 | 1.00  1.59 (1.32-1.93) | <0.0001 |
| **Smoking** | No | 1.00 |  | 1.00 |  |
|  | Yes | 1.58 (1.41-1.78) | <0.0001 | 1.78 (1.55-2.04) | <0.0001 |
| **Hypertension** | No  Yes | 1.00  1.57 (1.35-1.81) | <0.0001 | 1.00  1.50 (1.34-1.69) | <0.0001 |
| **Body mass index** | Non-obese | 1.00 |  | 1.00 |  |
|  | Obese | 1.47 (1.24-1.74) | <0.0001 | 1.24 (1.07-1.45) | 0.005 |
| **Alcohol consumption** | Non-moderate | 1.00 |  | 1.00 |  |
|  | Moderate | 0.86 (0.76-0.97) | 0.01 | 0.84 (0.74-0.95) | 0.007 |
| **Sleep disorder** | No  Yes | 1.00  1.27 (1.14-1.43) | 0.0001 | 1.00  1.28 (1.13-1.46) | 0.0001 |
| **Dyslipidemia** | No  Yes | 1.00  1.21 (1.06-1.37) | 0.003 | 1.00  1.18 (1.05-1.32) | 0.005 |
| **Physical activity** | No | 1.00 |  | 1.00 |  |
|  | Yes | 0.82 (0.73-0.91) | 0.0002 | 0.86 (0.67-1.09) | 0.21 |
| **Depression** | No  Yes | 1.00  1.17 (1.04-1.32) | 0.009 | 1.00  1.50 (1.18-1.90) | 0.0009 |

Time-independent predictive factors (gender, age, parental CVD) are not shown in the table but are included in the multi-adjusted regression models.

Risk of incident CVD factors according to the same factors not varying during follow-up - summary of multi-adjusted associations.

| **Predictive factors** | | **Incident factors** | | | | | | | | |
| --- | --- | --- | --- | --- | --- | --- | --- | --- | --- | --- |
|  |  | **Diabetes** | **Smoking** | **Hypertension** | **Obesity** | **Non-moderate**  **alcohol**  **consumption** | **Sleep**  **disorder** | **Dyslipidemia** | **Physical**  **inactivity** | **Depression** |
| **Diabetes** | No | - | NS | 1.00 | NS | NS | NS | 1.00 | NS | NS |
|  | Yes |  |  | 1.31 (1.01-1.69) |  |  |  | 1.23 (1.01-1.66) |  |  |
| **Smoking** | No | 1.00 | - | NS | 1.00 | NS | NS | 1.00 | 1.00 | 1.00 |
|  | Yes | 1.45 (1.25-1.68) |  |  | 1.32 (1.17-1.49) |  |  | 1.19 (1.09-1.30) | 1.39 (1.28-1.51) | 1.22 (1.11-1.34) |
| **Hypertension** | No  Yes | 1.00  1.92 (1.62-2.25) | NS | - | 1.00  1.84 (1.59-2.12) | NS | NS | 1.00  1.46 (1.30-1.63) | 1.00  1.17 (1.04-1.32) | NS |
| **Body mass index** | Non-obese | 1.00 | NS | 1.00 | - | NS | NS | 1.00 | 1.00 | NS |
|  | Obese | 3.92 (3.32-4.60) |  | 2.14 (1.87-2.43) |  |  |  | 1.14 (1.01-1.31) | 1.57 (1.36-1.80) |  |
| **Alcohol consumption** | Non-moderate | NS | NS | NS | 1.00 | - | NS | NS | NS | NS |
|  | Moderate |  |  |  | 0.87 (0.77-0.98) |  |  |  |  |  |
| **Sleep disorder** | No | NS | NS | 1.00 | NS | NS | - | 1.00 | NS | 1.00 |
|  | Yes |  |  | 1.13 (1.04-1.23) |  |  |  | 1.10 (1.01-1.18) |  | 1.77 (1.63-1.93) |
| **Dyslipidemia** | No  Yes | 1.00  1.76 (1.52-2.03) | NS | 1.00  1.28 (1.17-1.40) | 1.00  1.19 (1.04-1.35) | NS | NS | - | 1.00 | NS |
|  |  |  |  |  |  |  |  |  | 1.19 (1.08-1.30) |  |
| **Physical activity** | No | 1.00 | NS | 1.00 | 1.00 | NS | NS | 1.00 | - | 1.00  0.91 (0.84-0.99) |
|  | Yes | 0.78 (0.69-0.89) |  | 0.89 (0.82-0.96) | 0.69 (0.63-0.77) |  |  | 0.92 (0.85-0.99) |  |  |
| **Depression** | No | 1.00 | 1.00 | 1.00 | 1.00 | NS | 1.00 | 1.00 | 1.00 | - |
|  | Yes | 1.26 (1.09-1.46) | 1.16 (1.01-1.39) | 1.11 (1.02-1.20) | 1.22 (1.08-1.37) |  | 1.77 (1.61-1.94) | 1.12 (1.03-1.21) | 1.16 (1.07-1.26) |  |

HRs (95% CI). White background: p<0.05, light gray: p<0.01, middle gray: p<0.001, dark gray: p<0.0001, NS: non-significant. Time-independent predictive factors (gender, age, parental CVD) are not shown in the table but are included in the multi-adjusted regression models.

Risk of incident CVD factors according to the same factors varying during follow-up - summary of multi-adjusted associations.

| **Predictive factors** | | **Incident factors** | | | | | | | | |
| --- | --- | --- | --- | --- | --- | --- | --- | --- | --- | --- |
|  |  | **Diabetes** | **Smoking** | **Hypertension** | **Obesity** | **Non-moderate**  **alcohol**  **consumption** | **Sleep**  **disorder** | **Dyslipidemia** | **Physical**  **inactivity** | **Depression** |
| **Diabetes** | No | - | NS | 1.00 | NS | NS | NS | 1.00 | NS | 1.00 |
|  | Yes |  |  | 1.25 (1.04-1.49) |  |  |  | 1.48 (1.20-1.83) |  | 1.28 (1.03-1.59) |
| **Smoking** | No | 1.00 | - | 1.00 | NS | 1.00 | NS | 1.00 | 1.00 | 1.00 |
|  | Yes | 1.22 (1.01-1.47) |  | 0.86 (0.76-0.96) |  | 1.18 (1.04-1.34) |  | 1.12 (1.01-1.24) | 1.37 (1.24-1.50) | 1.22 (1.09-1.36) |
| **Hypertension** | No  Yes | 1.00  1.63 (1.42-1.88) | NS | - | 1.00  1.65 (1.45-1.87) | NS | NS | 1.00  1.48 (1.35-1.63) | 1.00  1.14 (1.03-1.25) | NS |
| **Body mass index** | Non-obese | 1.00 | NS | 1.00 | - | NS | NS | 1.00 | 1.00 | NS |
|  | Obese | 3.90 (3.38-4.51) |  | 2.24 (2.02-2.49) |  |  |  | 1.27 (1.12-1.44) | 1.64 (1.46-1.84) |  |
| **Alcohol consumption** | Non-moderate | NS | NS | NS | 1.00 | - | NS | NS | NS | NS |
|  | Moderate |  |  |  | 0.85 (0.75-0.96) |  |  |  |  |  |
| **Sleep disorder** | No | NS | NS | 1.00 | NS | NS | - | 1.00 | NS | 1.00 |
|  | Yes |  |  | 1.14 (1.04-1.25) |  |  |  | 1.19 (1.09-1.30) |  | 2.45 (2.25-2.68) |
| **Dyslipidemia** | No  Yes | 1.00  1.73 (1.51-1.97) | NS | 1.00  1.17 (1.08-1.28) | 1.00  1.18 (1.05-1.34) | NS | NS | - | 1.00 | NS |
|  |  |  |  |  |  |  |  |  | 1.17 (1.07-1.27) |  |
| **Physical activity** | No | 1.00 | NS | 1.00 | 1.00 | NS | NS | NS | - | 1.00  0.87 (0.76-0.99) |
|  | Yes | 0.82 (0.60-0.99) |  | 0.89 (0.77-0.99) | 0.56 (0.47-0.67) |  |  |  |  |  |
| **Depression** | No | NS | 1.00 | 1.00 | 1.00 | NS | 1.00 | 1.00 | 1.00 | - |
|  | Yes |  | 1.36 (1.01-1.84) | 1.16 (1.01-1.35) | 1.16 (1.01-1.42) |  | 2.39 (2.10-2.72) | 1.10 (1.01-1.27) | 1.27 (1.10-1.47) |  |

HRs (95% CI). White background: p<0.05, light gray: p<0.01, middle gray: p<0.001, dark gray: p<0.0001, NS: non-significant. Time-independent predictive factors (gender, age, parental CVD) are not shown in the table but are included in the multi-adjusted regression models.
