# Supplementary material for: A Global View of the Relationships between the Main Behavioural and Clinical Cardiovascular Risk Factors in the GAZEL Prospective Cohort
Source: PLoS One. 2016 Sep 6;11(9):e0162386. doi: 10.1371/journal.pone.0162386 (PMC5012694; doi:10.1371/journal.pone.0162386)
Supplement: Supplementary file 9 [file pone.0162386.s003.docx]

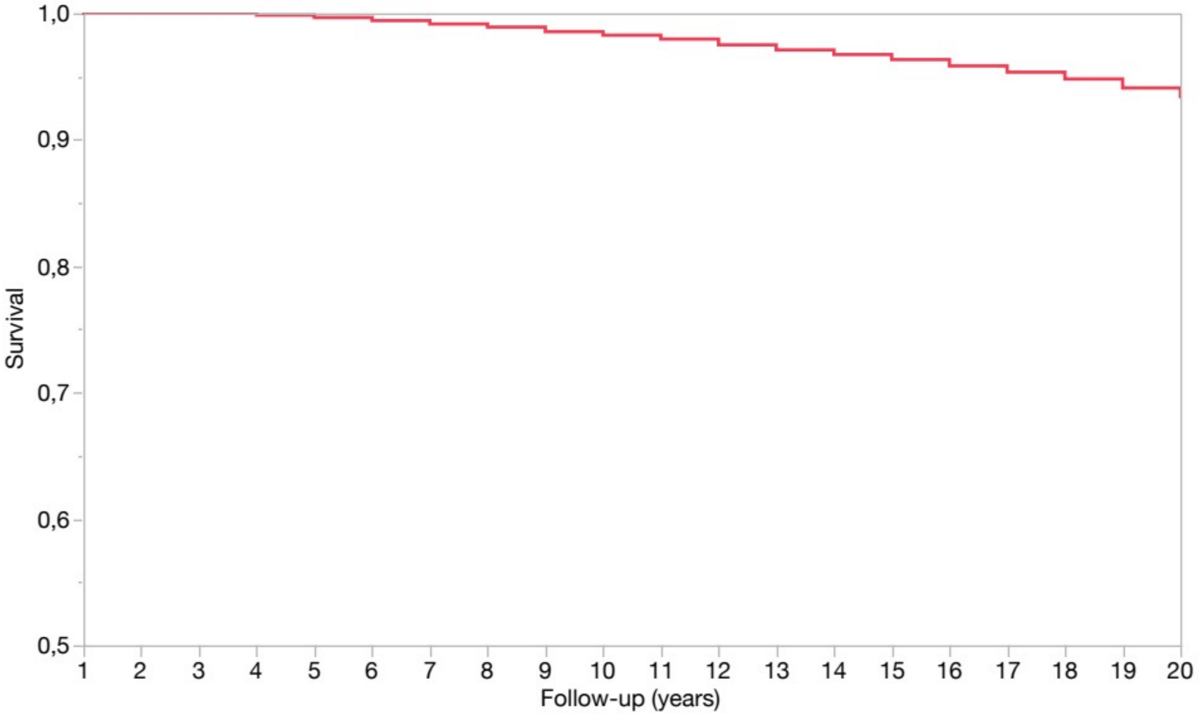
1,0
0,9
0,8
0,7
0,6
0,5
Survival
1 2 3 4 5 6 7 8 9 10 11 12 13 14 15 16 17 18 19 20
Follow-up (years)
